# Nuclear Myosin 1c Facilitates the Chromatin Modifications Required to Activate rRNA Gene Transcription and Cell Cycle Progression

**DOI:** 10.1371/journal.pgen.1003397

**Published:** 2013-03-21

**Authors:** Aishe Sarshad, Fatemeh Sadeghifar, Emilie Louvet, Raffaele Mori, Stefanie Böhm, Bader Al-Muzzaini, Anna Vintermist, Nathalie Fomproix, Ann-Kristin Östlund, Piergiorgio Percipalle

**Affiliations:** 1 Department of Cell and Molecular Biology, Karolinska Institute, Stockholm, Sweden; 2 Department of Cell Biology, The Wenner-Gren Institute, Stockholm University, Stockholm, Sweden; The University of North Carolina at Chapel Hill, United States of America

## Abstract

Actin and nuclear myosin 1c (NM1) cooperate in RNA polymerase I (pol I) transcription. NM1 is also part of a multiprotein assembly, B-WICH, which is involved in transcription. This assembly contains the chromatin remodeling complex WICH with its subunits WSTF and SNF2h. We report here that NM1 binds SNF2h with enhanced affinity upon impairment of the actin-binding function. ChIP analysis revealed that NM1, SNF2h, and actin gene occupancies are cell cycle-dependent and require intact motor function. At the onset of cell division, when transcription is temporarily blocked, B-WICH is disassembled due to WSTF phosphorylation, to be reassembled on the active gene at exit from mitosis. NM1 gene knockdown and motor function inhibition, or stable expression of NM1 mutants that do not interact with actin or chromatin, overall repressed rRNA synthesis by stalling pol I at the gene promoter, led to chromatin alterations by changing the state of H3K9 acetylation at gene promoter, and delayed cell cycle progression. These results suggest a unique structural role for NM1 in which the interaction with SNF2h stabilizes B-WICH at the gene promoter and facilitates recruitment of the HAT PCAF. This leads to a permissive chromatin structure required for transcription activation.

## Author Summary

Actin and myosin are key regulators of several processes that occur in the cell nucleus. In rRNA biogenesis, actin in complex with nuclear myosin 1c (NM1) is involved in several phases of rDNA transcription. Further, NM1 interacts with the chromatin remodelling complex WICH, with the subunits WSTF and SNF2h. The multiprotein assembly thus formed, termed B-WICH, is engaged in the post-initiation phase of pol I transcription. These observations have led to the proposal that the actin-NM1 interaction mediates the recruitment of the WICH complex to activate transcription. Recent evidence indicates that the WSTF component of the B-WICH complex facilitates SNF2h-dependent nucleosomes repositioning and remodels in this way the chromatin at the rRNA gene promoter. We show here that NM1 interacts with the WICH complex and that this interaction is required to establish permissive chromatin by promoting H3K9 acetylation. This mechanism leads to transcription activation and facilitates cell cycle progression. NM1 performs these actions by interacting with SNF2h, presumably stabilizing B-WICH at the gene promoter. We show also that NM1 is needed for association of the polymerase-associated actin with the rRNA gene. Actin and SNF2h compete for NM1 binding. Therefore we propose a two-step mechanism of gene activation where NM1 functions as a structural switch that connects pol I with chromatin for transcription activation and cell cycle progression.

## Introduction

Actin and myosin are involved in many nuclear functions in eukaryotic cells, including chromatin remodelling, transcription by all three RNA polymerases, biogenesis of ribonucleoprotein complexes and the repositioning of active gene loci [1]–[4]. There is evidence that actin interacts with the largest subunit of RNA polymerase I (pol I) and that nuclear myosin 1c (NM1) interacts with the pol I-specific transcription initiation factor TIF1a, in its phosphorylated form. NM1 is recruited in this way at the rRNA gene promoter before transcription initiation. These observations have led to the idea that actin and NM1 cooperate to assemble pol I at the gene promoter, and this leads to transcription initiation [5]–[7]. More recently, several further observations have led to the hypothesis that the actomyosin complex facilitates also the post-initiation phase of pol I transcription. These observations are that polymeric actin interacts with pol I, that controlled actin polymerization is required for transcription and that the NM1 ATPase cycle regulates association with the transcription machinery [6]–[9]. NM1, but not actin, is part of the multiprotein assembly B-WICH that contains the WICH chromatin remodeling complex with its subunits WSTF (Williams's syndrome transcription factor) and SNF2h [10]. B-WICH is also involved in the post-initiation phase of pol I transcription through a chromatin-based mechanism [10]. We have recently shown that WSTF, as a component of the WICH complex, is needed for SNF2h-mediated nucleosomes repositioning to remodel chromatin at the pol I promoter, a mechanism that leads to the association of histone acetyl transferases (HATs) with active gene promoters [10]–[12]. However, the precise contribution of NM1 as a component of B-WICH, and its potential role to generate permissive chromatin, have been matters of speculation [13].

Pol I transcription is arrested at entry into mitosis, while the nucleoli are dynamically disassembled. The nucleoli are reassembled around transcriptionally competent nucleolar organizer regions (NORs) at the end of cell division [14]. Pol I remains associated with NORs independently of ongoing transcription, whereas nucleolar processing proteins are recruited to newly synthesized rRNA after reactivation [14]–[17]. Chromatin is probably maintained in a relaxed configuration in active NORs to facilitate the association of factors involved in ribosome biogenesis [18], [19]. The mechanisms that establish and propagate the epigenetic state of rRNA genes require that an interplay occurs between DNA and histone-modifying enzymes that synergize with chromatin remodelling complexes and transcription machinery [20]. This interplay probably defines the transcriptional state of rDNA and prepares it for the rapid onset of transcription and nucleolar reformation as the cell exits mitosis. B-WICH remodels the chromatin of active rRNA genes, allowing HATs to associate [12]. We suggest, therefore, that B-WICH cooperates directly with pol I at the exit of mitosis for transcription activation, and that NM1 and actin, are instrumental for this crosstalk.

The considerations described above led us to postulate a model in which NM1 functions as a switch to facilitate the recruitment of the WICH chromatin remodeling complex during transcription activation [13]. This model provides the rationale for the present study. We set out to investigate how NM1 is involved in transcription activation as part of a complex with actin and as part of the multiprotein assembly B-WICH, which does not contain actin. We demonstrate that the interactions of NM1, SNF2h and actin with the rRNA gene are cell cycle-dependent. NM1, SNF2h and actin associate with the rDNA at the exit from mitosis only when pol I transcription has been reactivated. Furthermore, by interacting with SNF2h in a manner that is dependent on the NM1 motor function, we show that NM1 has a primary role in promoting PCAF-mediated H3K9 acetylation at the gene promoter. This acetylation allows transcription activation and cell cycle progression. The present findings lead us to propose a key structural role for NM1 in connecting the pol I machinery with chromatin remodeling during transcription activation.

## Results

### The rRNA gene occupancies of NM1, actin, and SNF2h are cell cycle-dependent

**Figure 1:**
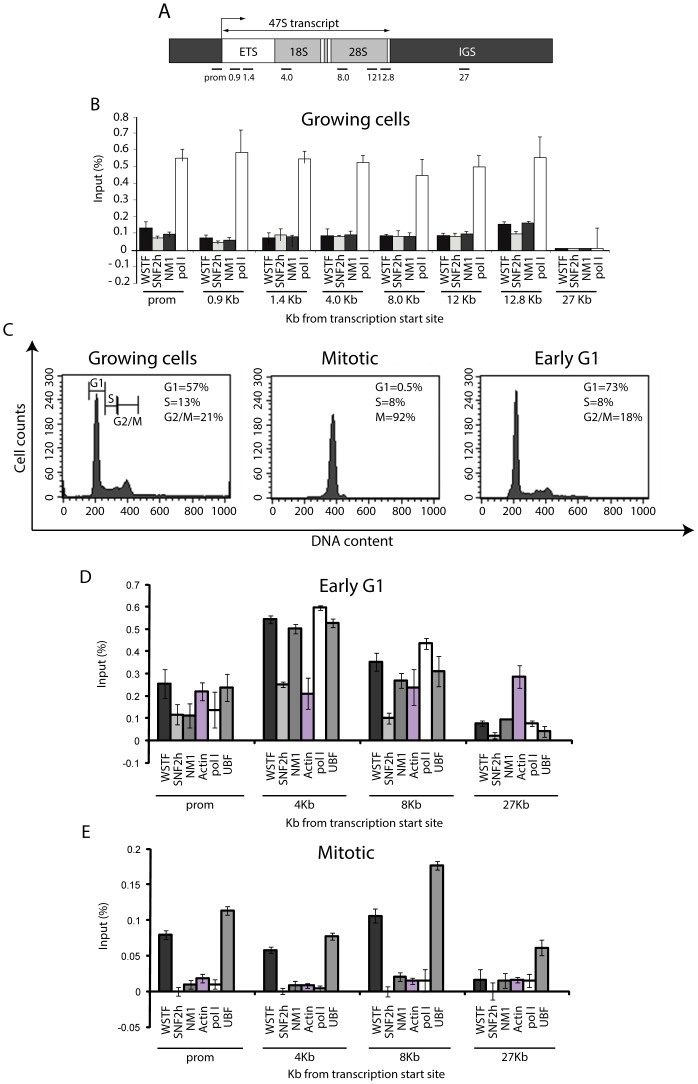
rRNA gene occupancy of NM1, SNF2h, and actin is cell cycle-dependent. (A) Schematic representation of the human rDNA transcription unit. (B) ChIP and qPCR on growing HeLa cells with antibodies as indicated, detected all along the human rDNA transcription unit. The values are presented as the percentages of the input signal for each primer pair. Error bars represent standard deviations. (C) Cell cycle profiles monitored by FACS analysis on growing HeLa cells, on nocodazole-blocked HeLa cells and on HeLa cells 2 h after release from the nocodazole block. (D) ChIP and qPCR on HeLa cells in early G1 at the rRNA gene promoter, 18S rDNA, 28S rDNA and IGS with antibodies as indicated below the bars. The values are presented as the percentage of the input signal for each primer pair. (E) ChIP and qPCR on mitotic HeLa cells blocked in prometaphase at the rRNA gene promoter, 18S and 28S rDNA as well as IGS with antibodies indicated below the bars. The values are presented as the percentage of the input signal for each primer pair.

NM1 and actin associate with rDNA promoters and coding regions [7], [10], and WSTF and SNF2h associate in a similar distribution [10]. Figure 1 shows chromatin immunoprecipitations (ChIP) and quantitative real-time PCR (qPCR) results that confirm that NM1, WSTF and SNF2h bind promoter (prom), externally transcribed sequences (ETSs) (0.9 Kb, 1.4 Kb), 18S rDNA (4 Kb,), and 28S rDNA (8 Kb, 12 Kb, 12.8 Kb) but not intergenic sequences (IGS) (27 Kb) (see Figure 1A for the location of the individual primers; Figure 1B). This distribution reflects the NM1 distribution along the entire rDNA unit observed in a previous study [8], except for the IGSs which appear to be devoid of NM1 (Figure 1B).

We next examined the rRNA gene associations of actin, NM1, WSTF and SNF2h in mitotic cells, when pol I transcription is arrested concomitantly with nucleolar disassembly. To start addressing this question, we synchronized cells in prometaphase using 40 ng/ml nocodazole. Following the nocodazole block, cells were harvested and released from the block, allowing them to pass through all mitotic stages. The cell cycle dynamics of the nocodazole block and the release were quantitatively characterized by flow cytometry (Figure 1C). Just after release of the nocodazole block (t = 0 min), 92% of the cell population was in prometaphase, whereas 2 h after release from the block (t = 120 min), the number of mitotic cells had decreased to 5% of the total population, the majority of cells being in G1 (73%) (Figure 1C; Figure S1). To determine whether the gene associations of actin, NM1, WSTF and SNF2h with rDNA are dependent on cell cycle, we performed ChIP on chromatin isolated from mitotic (t = 0 min) and early G1 cells (t = 120 min). In these experiments, formaldehyde-crosslinked chromatin was subjected to immunoprecipitations with antibodies to WSTF, SNF2h, NM1 and actin, as well as with antibodies to the pol I and pol I-specific transcription factor UBF (upstream binding factor). The precipitated chromatin was analyzed by qPCR with primers amplifying the rRNA gene promoter, 18S rDNA, 28S rDNA and IGS. Consistent with an active role in transcription, the gene promoter, 18S and 28S rDNAs were precipitated from chromatin isolated from early G1 cells (t = 120 min) with antibodies to actin, NM1, WSTF and SNF2h (Figure 1D). In contrast, when analyzing chromatin from mitotic cells at t = 0 min, we detected a massive drop in the amount of promoter and transcribed regions that were precipitated with the anti-NM1 antibodies, and levels below detection in the case of SNF2h antibodies (Figure 1E). In mitotic cells, rDNA was also devoid of actin (Figure 1E). Remarkably, WSTF antibodies, like the antibodies against UBF, precipitated promoter and transcribed regions from chromatin that had been isolated from both early G1 and mitotic cells, although with slightly lower efficiency (Figure 1D–1E). When analyzing the 3′ end of the rRNA gene, we detected a general decrease in the amount of intergenic regions precipitated from mitotic chromatin with SNF2h, NM1 and actin antibodies in comparison to early G1 chromatin (Figure 1D–1E). A similar scenario was detected with pol I but not with UBF antibodies (Figure 1D–1E). We do not know why in contrast to interphase chromatin, at early G1 WSTF, NM1, SNF2h and actin occupy IGS sequences together with pol I. It may be due to intrinsic local differences between interphase and early G1 chromatin. Anyhow, similarly to both pol I and UBF, NM1, WSTF and SNF2h colocalized with sites in nucleoli at which FUrd (fluorine-conjugated UTP analogue) had been incorporated in telophase/early G1 cells (see Figures S2, S3). We conclude that NM1, SNF2h and actin occupy rRNA genes in interphase and are released in mitotic cells (see also Figure S4), to re-associate only at telophase/early G1 when transcription is activated. In contrast, WSTF association with rRNA gene is independent of the cellular stage, consistent with earlier studies on the association of WSTF with mitotic chromosomes [21].

**Figure 2:**
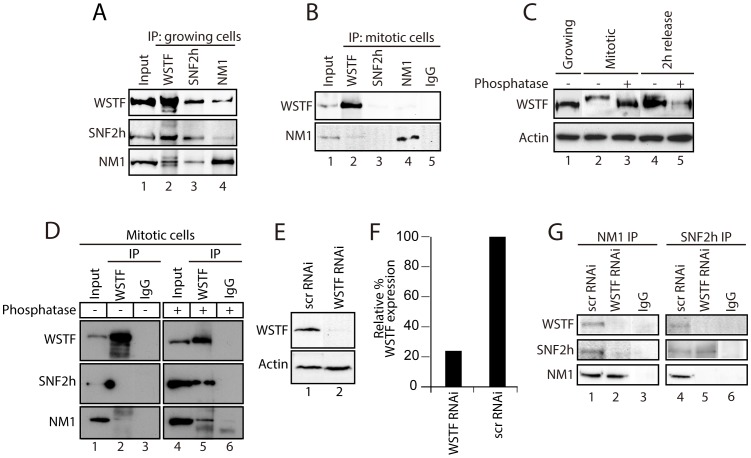
During cell division, WSTF phosphorylation events govern the assembly and disassembly of B-WICH. (A) NM1, SNF2h and WSTF are co-precipitated from protein extracts prepared from growing HeLa cells. Bound proteins were detected on immunoblots. 10% of the input is shown in Lane 1. IP, immunoprecipitation. (B) Co-precipitations of NM1, SNF2h and WSTF are impaired from protein extracts prepared from mitotic HeLa cells at t = 0 min from the nocodazole block. Bound proteins were detected on immunoblots. 10% of the input is loaded in Lane 1. IP, immunoprecipitation. (C) WSTF becomes phosphorylated at the onset of mitosis. Lysates were prepared from growing HeLa cells (Lane 1), mitotic HeLa cells at t = 0 min from the nocodazole block (Lanes 2 and 3) or HeLa cells after t = 120 min release from the block (Lanes 4 and 5). Where indicated extracts were subjected to phosphatase treatment (Lanes 3 and 5). In all cases lysates were separated by 7% phospho-affinity SDS-PAGE and analyzed on immunoblots for WSTF and actin. (D) Co-precipitations of WSTF, SNF2h and NM1 are dependent on WSTF phosphorylation. Mitotic extracts from HeLa cells at t = 0 min from the nocodazole block, untreated (Lanes 1–3) or treated with phosphatase (Lanes 4–6), were subjected to immunoprecipitations with anti-WSTF antibodies or non-specific IgGs. Bound proteins were separated by SDS PAGE and analyzed on immunoblots for WSTF, SNF2h and NM1. 5% of the input is shown in Lane 1. IP, immunoprecipitation. (E–F) WSTF steady state expression levels on immunoblots of lysates prepared from control (scrRNAi) and WSTF-silenced HeLa cells. Right panel, densitometric quantification of WSTF steady state protein expression relative to actin. (G) In growing HeLa cells, co-precipitations of NM1 and SNF2h are impaired as a consequence of WSTF gene knockdown by siRNA (WSTF RNAi) but not when cells are subjected to control experiments with scrambled RNAi oligonucleotides (scrRNAi). In Lanes 1 and 4, 20% of the input material was loaded.

To correlate the different patterns of gene occupancy observed for NM1, SNF2h and WSTF with the assembly of B-WICH, we prepared protein extracts from mitotic HeLa cells blocked with nocodazole (t = 0 min). The mitotic extracts were subjected to immunoprecipitations with antibodies to NM1, WSTF and SNF2h. The results in Figure 2 show that WSTF, SNF2h and NM1 did not co-precipitate, indicating that the strong association observed in growing cells is lost upon entry into mitosis (Figure 2A and 2B). WSTF is phosphorylated by MAPK [22]. To find out whether the loss of tight association is due to WSTF phosphorylation, we resolved extracts from growing or mitotic cells by phosphate affinity SDS PAGE [23]. Analysis of immunoblots for WSTF revealed specific gel retardation in the case of mitotic extracts, which could not be detected 2 h after release from the nocodazole block or upon phosphatase treatment (Figure 2C). Phosphatase-treated mitotic extracts were next subjected to immunoprecipitations with WSTF antibodies. Under these conditions, analysis of the bound proteins on immunoblots showed that NM1 and SNF2h were specifically co-precipitated with WSTF, compared to untreated mitotic extracts (Figure 2D). Even though we do not know the precise mechanism, we conclude that reversible phosphorylation events during mitosis specifically target WSTF and prevent co-precipitation of the B-WICH components. To determine whether WSTF is required for the association of NM1 and SNF2h, we knocked down the WSTF gene in HeLa cells by RNAi (Figure 2E–2F), under which conditions the rRNA synthesis is down-regulated [10]. Nuclear extracts prepared from WSTF-silenced cells or cells treated with control scrambled RNAi (scrRNAi) oligonucleotides were next subjected to immunoprecipitations with anti-NM1 and anti-SNF2h antibodies. Bound proteins were analyzed on immunoblots for NM1, SNF2h and WSTF. We found that co-precipitations of NM1 and SNF2h from nuclear extracts prepared from WSTF-silenced cells were impaired in comparison to control cells (Figure 2G). These observations show that WSTF is important for the assembly of NM1 and SNF2h within the B-WICH complex.

In summary, NM1 and SNF2h, but not WSTF, associate with rDNA in a cell cycle-dependent manner. The results presented above show that phosphorylation events that specifically target WSTF during cell division drastically affect the stability of the B-WICH complex and probably lead to B-WICH disassembly by hindering specific protein-protein interactions. These phosphorylation events are reversible, and it is possible that they are impaired at the exit of mitosis, when WSTF contributes to B-WICH assembly on active rRNA genes.

### A functional NM1 modulates SNF2h and actin association with active rRNA gene

**Figure 3:**
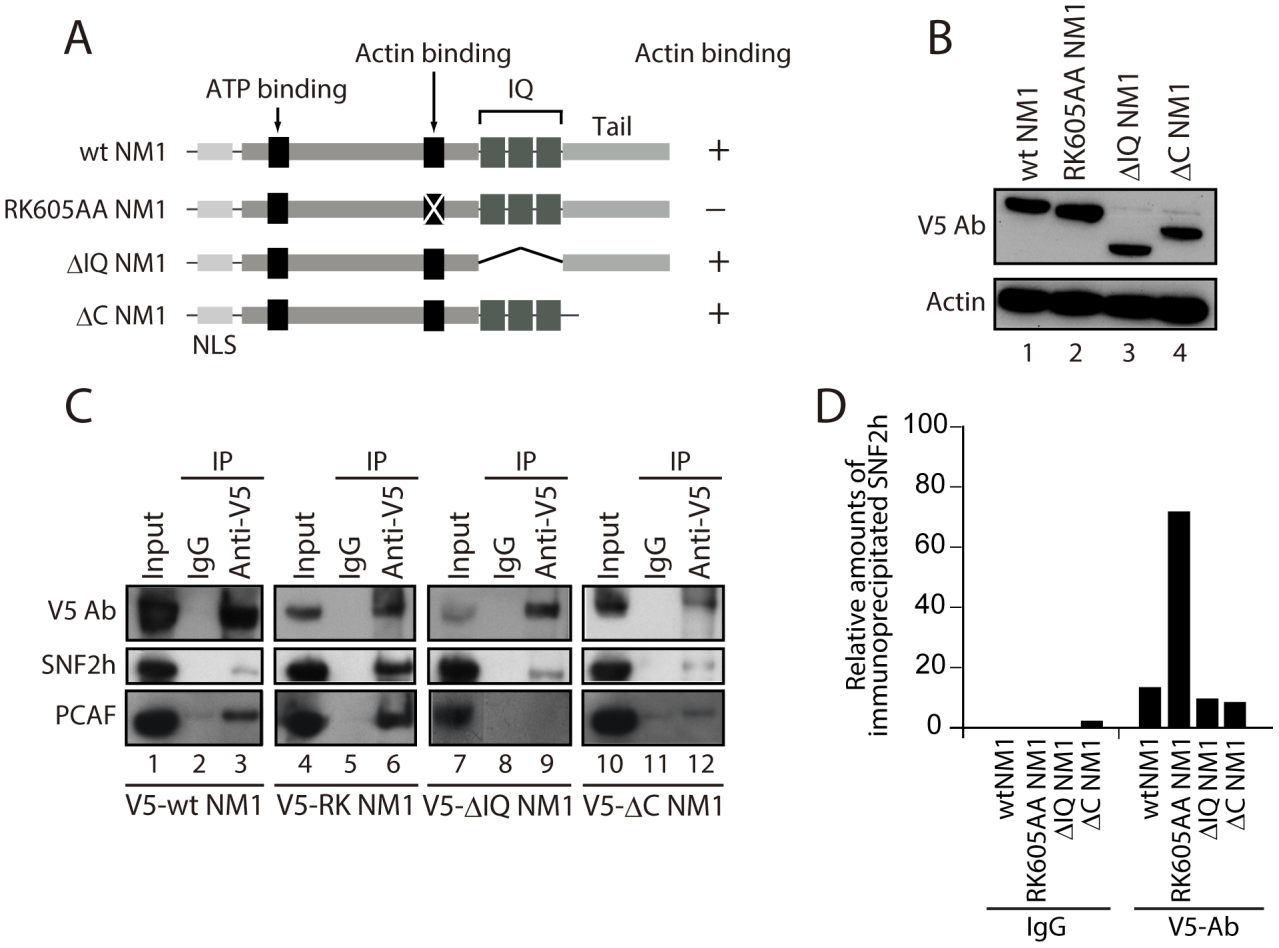
NM1 interacts with SNF2h and the HAT PCAF. (A) Schematic representation of V5-tagged wt and mutated NM1 constructs stably expressed in HEK293T cell lines. (B) Steady state expressions of V5-tagged wt and mutated NM1 constructs were monitored on immunoblots for V5 and actin antibodies as loading control. (C) Co-precipitations of SNF2h and PCAF from total lysates obtained from HEK293T cells stably expressing wt and mutated V5-tagged NM1 constructs as indicated. 10% of the input is shown in Lane 1. IP, immunoprecipitation. (D) Semiquantitative densitometric analysis of endogenous SNF2h levels co-precipitated with V5-tagged NM1 constructs relative to input.

We next evaluated the potential contribution of NM1 to B-WICH assembly on the rDNA by studying how NM1 associates with the WICH subunits and with the rRNA gene. For this purpose we used HEK293T cell lines, which stably express V5-tagged wild-type NM1 (V5-wtNM1), and a V5-tagged NM1 point mutant that has an impaired actin-binding function and thus an impaired motor activity (V5-RK605AA NM1) [8]. We also used HEK293T cells stably expressing V5-tagged deletion constructs that lack the C-terminal calmodulin binding IQ motifs (V5-ΔIQ NM1) or the tail domain (V5-ΔC NM1) [8] (see Figure 3A). Steady state expression of all constructs was monitored on immunoblots of total cell lysates with an anti-V5 antibody (Figure 3B). We next subjected total lysates from each of the above cell lines to immunoprecipitations with the V5 antibody. As expected, the RK605AA NM1 mutant specifically lost the ability to bind to actin in comparison to V5-tagged wild-type NM1 or deletion NM1 mutants lacking the C-terminus (Figure S5A–S5B). SNF2h was co-precipitated by all V5-tagged NM1 constructs but, interestingly, increased levels of co-precipitated SNF2h were detected in the fraction bound to the V5-RK605AA NM1 mutant (Figure 3C–3D). Finally, consistent with evidence that the HAT PCAF interacts with the B-WICH complex [12], PCAF was co-precipitated with full-length V5-tagged wt NM1, as well as with the RK605AA NM1 mutant (Figure 3C–3D). However, PCAF was not efficiently co-precipitated with either of the C-terminally deleted NM1 constructs, V5-ΔIQ NM1 and V5-ΔC NM1 (Figure 3C, Lanes 8, 9 and Lanes 11, 12). SNF2h, PCAF and V5 were not detected in mock experiments in which the lysates were incubated with non-specific immunoglobulins (IgGs), supporting the specificity of the immunoprecipitation assays (Figure 3C). Furthermore, densitometric quantifications of bound SNF2h over two independent measurements showed a specific increase in the amount of endogenous SNF2h co-precipitated with V5-RK605AA NM1 (Figure 3D). These results suggest that NM1 interacts directly with SNF2h, and that the interaction is enhanced when actin binding is impaired. In addition, the NM1 C-terminal deletion mutants V5-ΔIQ and V5-ΔC NM1 failed to co-immunoprecipitate with PCAF, showing that PCAF targets the NM1 C-terminus.

**Figure 4:**
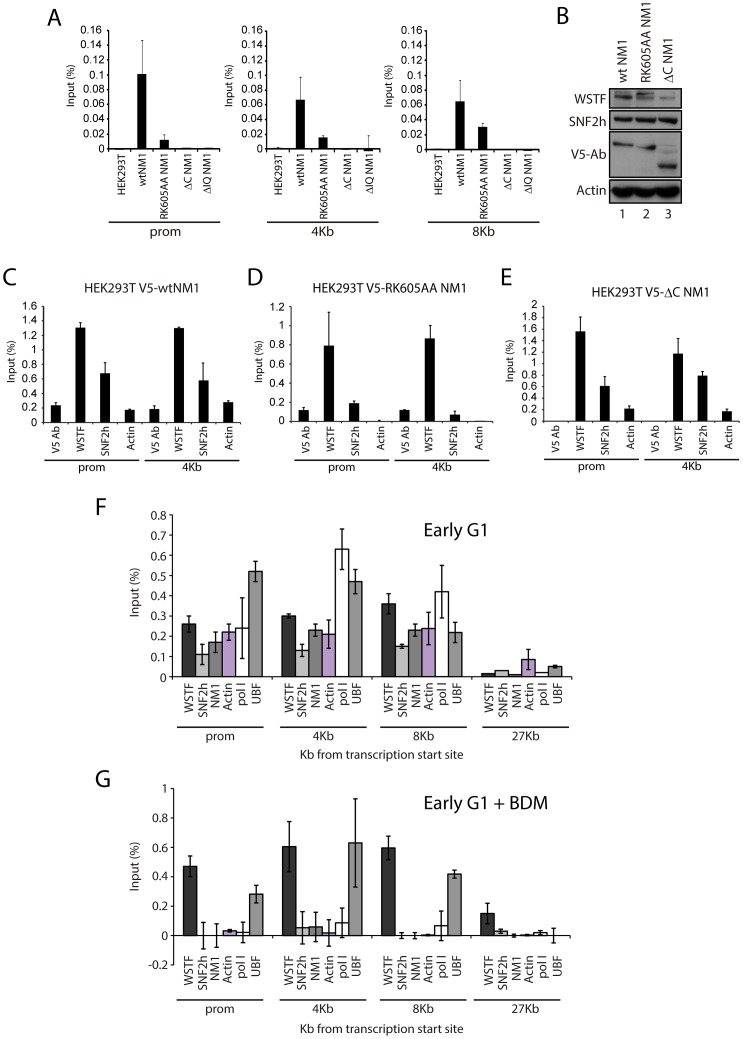
NM1 C-terminus and intact motor function are required for the association of NM1, SNF2h, and actin with rRNA genes. (A) ChIP assays performed on HEK293T cells or HEK293T cells constitutively expressing V5-tagged wt and mutated NM1 constructs (as indicated) were analyzed by qPCR. The bars diagrams show the relative amounts of rDNA promoter, 18S rDNA and 28S rDNA precipitated with V5 antibodies. The values are presented as the percentage of the input signal for each pair. Error bars represent standard deviations. (B) Steady state expression levels of endogenous WSTF and SNF2h analyzed on immunoblots of total cell lysates from HEK293T cells expressing V5-wtNM1, V5-RK605AA NM1 and V5-ΔC NM1 mutants. WSTF and SNF2h steady state expression levels were normalized against endogenous actin levels. Expressions of V5-wtNM1, V5-RK605AA NM1 and V5-ΔC NM1 mutants were monitored with anti-V5 antibodies. (C–E) ChIP assays on chromatin from HEK293T cells stably expressing V5-wtNM1, V5-RK605AA and V5-ΔC NM1 mutants with antibodies against V5, WSTF, SNF2h and actin. qPCR analysis was performed with primers amplifying promoter and 18S rDNA. The values are presented as the percentage of the input signal for each pair. Error bars represent standard deviations. (F–G) ChIP assays on chromatin from HeLa cells untreated (F) or treated with BDM (G) using the indicated antibodies (see below the bars). qPCR analysis was performed with primers amplifying rRNA gene promoter, 18S and 28S rDNA as well as IGS. The values are presented as the percentage of the input signal for each primer pair. Error bars represent standard deviations.

To investigate how NM1 interacts with rDNA we applied ChIP using an anti-V5 antibody on crosslinked chromatin isolated from the same HEK293T cell lines used above which stably express the V5-tagged NM1 constructs. qPCR analysis on the precipitated chromatin fragments with primers amplifying the promoter, 18S or 28S rDNA confirmed that the V5-RK605AA NM1 mutant associates less strongly with the rRNA gene than wtNM1 (Figure 4A; Figure S5C–S5D) [8]. However, neither V5-ΔIQ NM1 nor V5-ΔC NM1 efficiently precipitated rRNA gene promoters or transcribed regions (Figure 4A; Figure S5C–S5D). Earlier in vitro studies suggested that the recombinantly expressed NM1 C-terminal tail domain interacts with single-stranded DNA [1]. Therefore, taken altogether, our results show that NM1 specifically binds rDNA via its C-terminus. Interestingly, in the cells stably expressing the V5-tagged RK605AA NM1 mutant where WSTF, SNF2h and actin showed unaltered steady state expressions (Figure 4B), we observed drops in SNF2h and actin levels of occupancy at gene promoters and 18S (Figure 4C–4D). In contrast, in cells stably expressing the C-terminal NM1 deletion construct that does not interact with chromatin, actin and SNF2h gene occupancies were only marginally affected (Figure 4C–4E). Remarkably, the rRNA gene occupancy of PCAF was impaired in the cells that expressed the V5-ΔC NM1 mutant, and it was marginally affected on expression of the V5-RK605AA NM1 mutant (see Figure S6).

HeLa cells synchronized in early G1 obtained 2 h after release from the nocodazole block next treated with butane dione monoxime (BDM), a cell permeable drug that impairs ATPase activity, pushing the equilibrium towards low-affinity actomyosin complexes and affecting actin dynamics [24]. Under these conditions, we compared the rRNA gene occupancies of endogenous actin, NM1, WSTF, SNF2h with those of pol I and UBF, using ChIP and qPCR analysis. BDM treatment led to the specific depletion of actin, NM1, SNF2h and pol I from rRNA gene promoters, transcribed regions and intergenic regions (Figure 4F–4G) from the corresponding levels in untreated cells, whereas WSTF and UBF levels in the same regions were not significantly affected (Figure 4F–4G). These observations suggest that the actin-binding function and the motor activity of NM1 contribute to stabilizing the association of actin and SNF2h with active rRNA genes while the NM1 C-terminus is indispensable for the association of PCAF with the rDNA. In contrast, NM1 is not required for the association of WSTF with the gene.

### NM1 activates pol I transcription and cell cycle progression by mediating H3K9 acetylation

**Figure 5:**
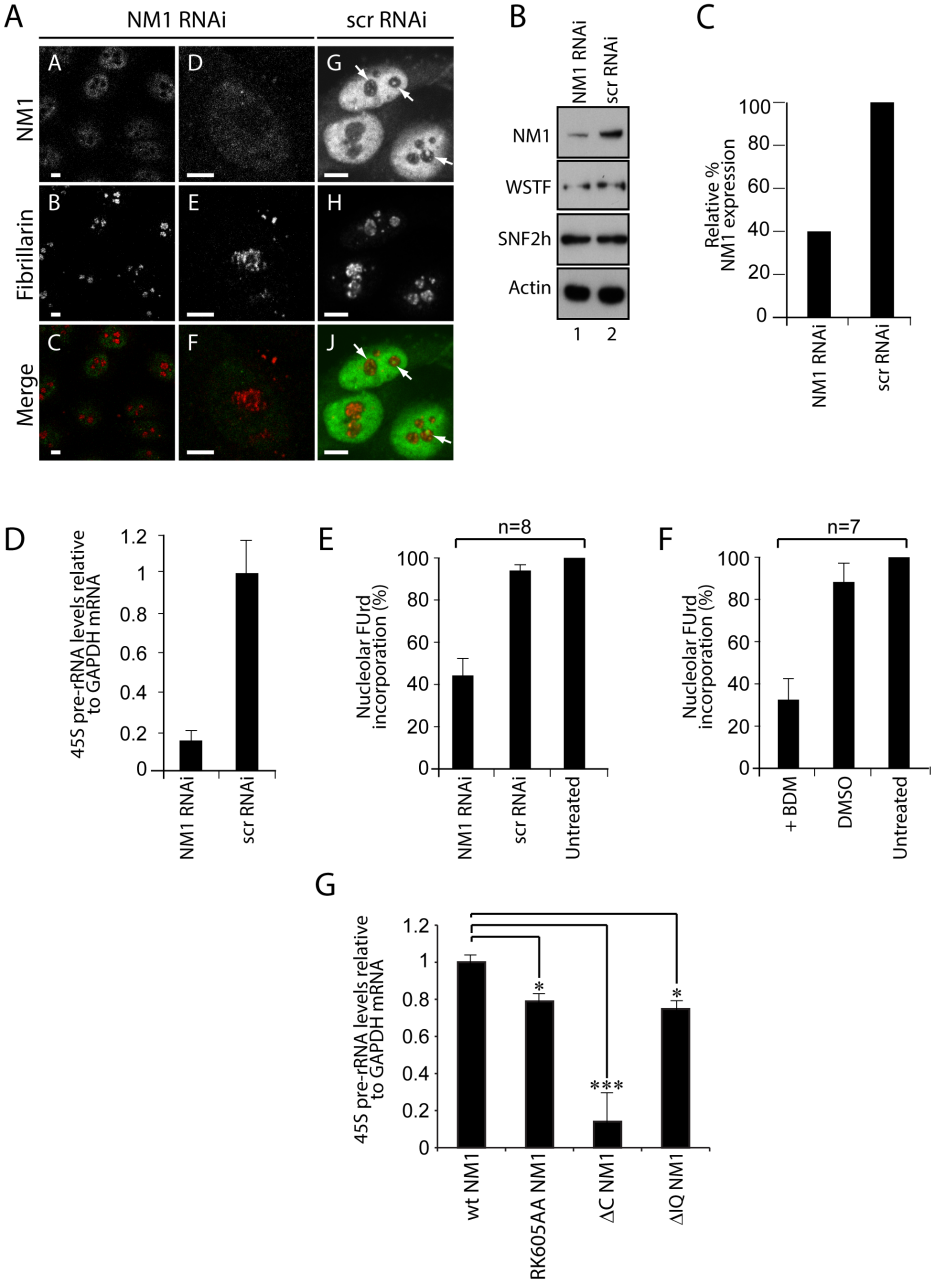
A functional NM1 is required for the activation of pol I transcription. (A) RNAi-mediated NM1 gene knockdown in HeLa cells analyzed by double immunostaining and confocal microscopy with antibodies to NM1 and fibrillarin, after transfection of NM1-specific siRNA or control oligos (scrRNAi). Scale bars, 5 µm. (B) Steady state expression levels for NM1, WSTF, SNF2h and actin monitored on immunoblots of total lysates prepared from HeLa cells subjected to NM1 gene knockdown (NM1 RNAi) or from cells subjected to scrRNAi. (C) Semiquantitative densitometric quantification of NM1 steady state protein expression relative to actin. (D) qRT-PCR analysis of 45S pre-rRNA performed on total RNA prepared from HeLa cells subjected to NM1 gene knockdown (NM1 RNAi) or from cells subjected to control siRNA oligonucleotides (scrRNAi). The 45S pre-rRNA levels are relative to GAPDH mRNA. (E–F) FUrd incorporation assays on living HeLa cells subjected to (E) NM1 gene knockdown by RNAi, or (F) treated with BDM. Transcription was monitored by short FUrd pulses to follow incorporation into nascent nucleolar transcripts. Quantification of the FUrd foci after immunostaining and confocal microscopy was performed by measurements on randomly selected nucleolar regions in the images. The signal was quantified using ImageJ software. The average of the mean grey values in control cells was determined, and defined as hundred percent of signal. The average of the mean grey values measured after treatment was expressed proportionally. n = number of cells in each experiment. Error bars represent standard deviations. (G) rRNA synthesis in HEK293T cells stably expressing V5-wtNM1, V5-RK605AA, V5-ΔC or V5-ΔIQ NM1 mutants. For the analysis, relative 45S pre-rRNA levels were monitored from total RNA preparations by RT–qPCR using GAPDH mRNA as internal control. Error bars represent the standard deviation of three independent experiments. Significances [*p*_RK605AA NM1_ = 0.019 (*), *p*_ΔC NM1_ = 0.0006 (***), *p*_ΔIQ NM1_ = 0.05 (*)] were obtained by Student's T-test, two-sample, equal variance.

The above findings support the idea that NM1 is involved in transcription activation and has an impact on cell cycle progression. We subjected subconfluent asynchronous HeLa cells to RNAi-mediated NM1 gene knockdown, as previously described [7], in order to investigate this idea (see Figure 5A–5C). We next isolated total RNA from NM1-silenced cells or from cells subjected to control RNAi experiments with scrambled RNAi oligonucleotides (scrRNAi) and measured relative pre-rRNA levels by quantitative real-time reverse transcription PCR (qRT-PCR). Using primers amplifying 45S pre-rRNA, we detected a specific fivefold drop in the amount of nascent transcript following NM1 gene knockdown, relative to GAPDH mRNA levels (Figure 5D). These findings suggest that NM1 plays an important role in pol I transcription activation. This point was corroborated by FUrd incorporation assays performed on living cells subjected to NM1 gene knockdown. In these experiments, we performed short pulse chases of 5–8 min to allow selective incorporation of the cell-permeable FUrd into nascent rRNA transcripts. This allowed to monitor primarily the nucleolar transcripts, as previously described [25], [26]. As revealed by immunofluorescence and confocal microscopy, the amount of FUrd incorporated into nucleolar transcripts decreased by 40–50%, concomitantly with decreased steady state expression of NM1 (Figure 5E; Figure S7). A similar 40–50% drop in the levels of FUrd incorporated into nascent nucleolar transcripts occurred following BDM treatment performed on the living cells (Figure 5F; Figure S7). If a functional NM1 is important for pol I transcription activation and affects actin and SNF2h occupancy, pol I transcription should be down-regulated in the cells stably that stably express NM1 mutants that cannot interact with actin or with chromatin. Indeed, analysis of the relative levels of 45S pre-rRNA by qRT-PCR on total RNA isolated from HEK293T cells stably expressing V5-RK605AA NM1, V5-ΔC NM1 and V5-ΔIQ NM1 mutants showed a significant decrease in pol I transcription in comparison to wild-type (Figure 5G). The above findings confirm that NM1 plays a primary role in pol I transcription. Our results also show, for the first time, that NM1 probably plays its role in pol I transcription in a complex with actin and bound to the chromatin.

**Figure 6:**
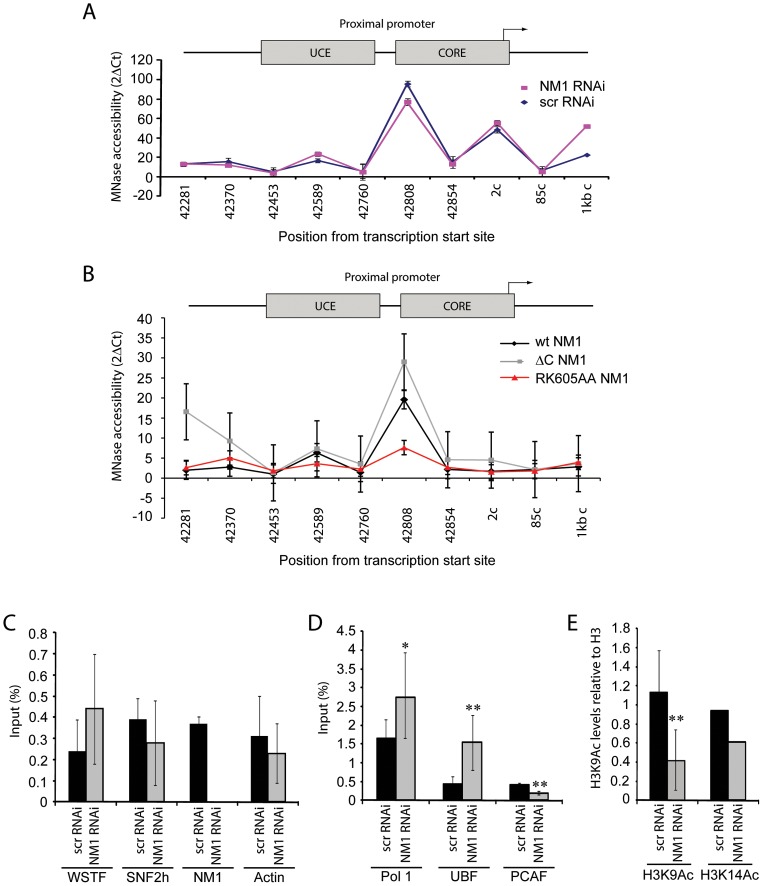
NM1 controls the levels of H3K9 acetylation for the activation of pol I transcription. (A) Chromatin profile from NM1 knockdown cells (NM1 RNAi, red line) and control cells (Control scrRNAi, blue line) shown as 2ΔCt of undigested and MNase digested cross-linked chromatin. The position of each primer pair used is given below the graph; 2c (coding) denotes Position 2 in the coding region. Error bars represent standard deviations of three separate experiments. (B) Chromatin profile from HEK293T cells stably expressing V5-wtNM1, V5-RK605AA NM1 or V5-ΔC NM1 shown as 2ΔCt of undigested and MNase digested cross-linked chromatin. The position of each primer pair is indicated below the graph; 2c (coding), Position 2 in the coding region. Error bars represent standard deviations of three separate experiments. (C–E) ChIP and qPCR analysis on chromatin isolated from NM1 knockdown cells (NM1 RNAi) and control cells (scrRNAi), (C) using antibody against WSTF, SNF2h, NM1 and actin, (D) antibodies to pol I, UBF or PCAF, and (E) antibodies against histone H3 acetylated on K9 (H3K9Ac) or histone H3 acetylated on K14 (H3K14Ac). In all cases, qPCR analysis was performed with primers amplifying rRNA gene promoters. The values are presented as the percentage of the input signal for each primer pair. Error bars represent standard deviations. Significances [(*), *p* = 0.05 and (**), *p* = 0.02] were obtained by Student's T-test, two-sample equal variance.

To elucidate whether the contribution of NM1 to transcription activation is through a direct effect on the chromatin, we silenced the NM1 gene by RNAi methods and analyzed potential changes in rDNA chromatin upstream of the transcription start site (21 kb to +300 kb) using a high-resolution MNase assay performed on cross-linked chromatin [27]. We found that NM1 gene knockdown caused a degree of chromatin protection over the rRNA gene promoter, including the upstream control element (UCE) and the core promoter element (CORE) (Figure 6A). This chromatin protection was enhanced in cells that stably express the V5-RK605AA NM1 mutant above the level of V5-wtNM1 cells. In contrast, chromatin accessibility increased following stable expression of the NM1 C-terminal mutants, V5-ΔC NM1 and V5-ΔIQ NM1 (Figure 6B; Figure S8). Our interpretation is that the V5-RK605AA NM1 mutant hinders assembly of the chromatin remodeling complex, possibly by sequestering SNF2h. In contrast, the NM1 C-terminal mutants are not likely to obstruct WICH assembly, since they do not disturb the gene occupancy of SNF2h and, consistently, we observed that stable expression of V5-ΔC NM1 or V5-ΔIQ NM1 led to increased chromatin accessibility. V5-RK605AA NM1, V5-ΔC NM1 and V5-ΔIQ NM1 are all negative regulators of rRNA synthesis. Therefore we conclude that a functional NM1 activates transcription through a chromatin-based mechanism.

We applied ChIP and qPCR analysis on chromatin isolated from NM1-silenced cells and analyzed the occupancies of WSTF, SNF2h, actin and components of the pol I machinery at the rRNA gene promoter, in order to obtain further mechanistic insights. At the gene promoter, we found significantly increased pol I and UBF occupancies concomitantly with a drop in the levels of the HAT PCAF (Figure 6D), compared to WSTF, SNF2h and actin levels (Figure 6C). H3K9 acetylation levels were lower under the same conditions (Figure 6E). Considering that a functional NM1 is required for rRNA synthesis, increased pol I levels at gene promoters in NM1-silenced cells are likely to be the result of polymerase stalling, due to local changes in chromatin composition. B-WICH-mediated chromatin remodeling leads to a local deposition of HATs at the rRNA gene promoter [12]. Our results suggest that NM1 contributes to pol I transcription activation by modulating the assembly of B-WICH and facilitating the recruitment of PCAF at the gene promoter to maintain H3K9 acetylation levels.

**Figure 7:**
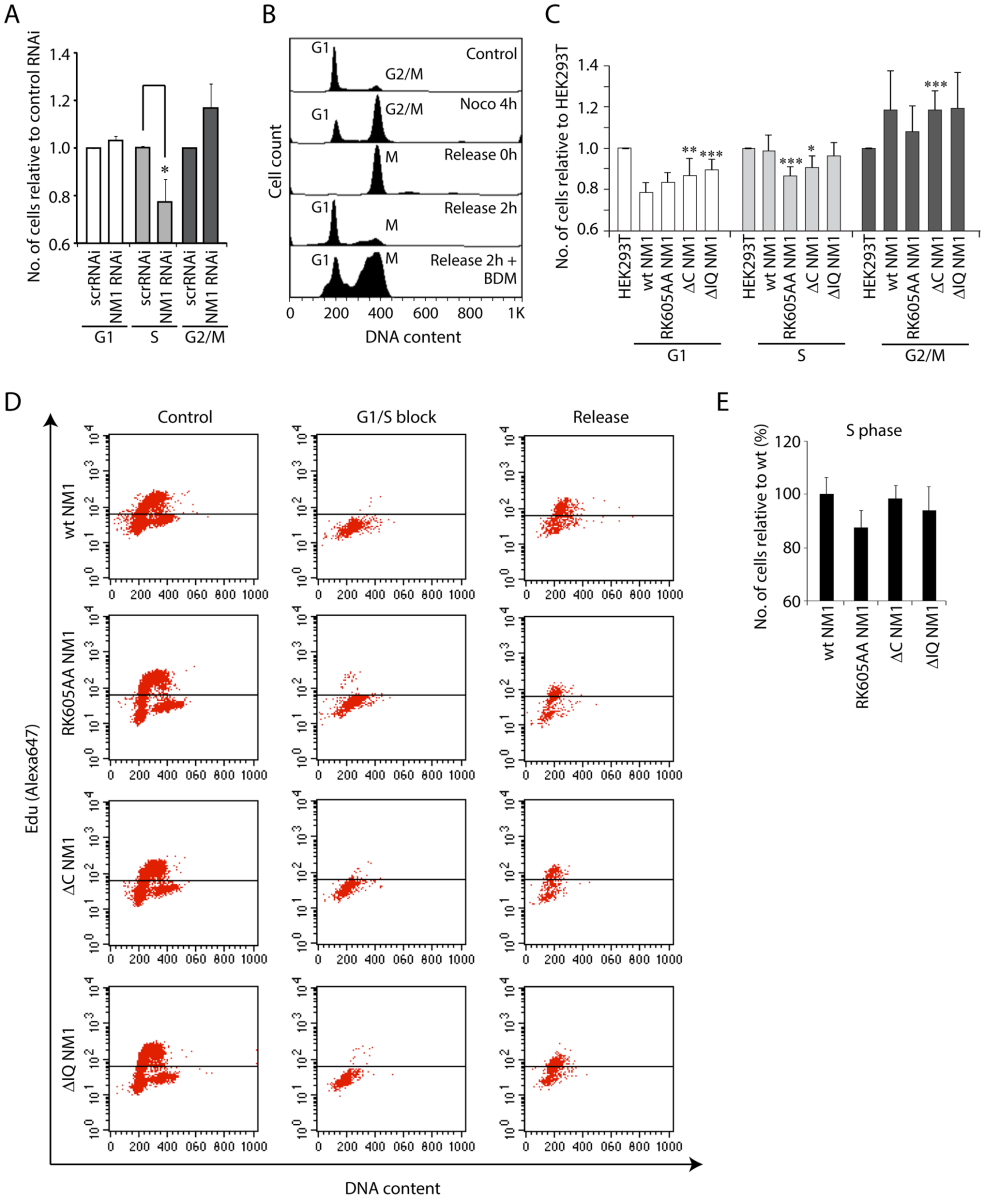
NM1 gene silencing by RNAi leads to a delay in cell cycle progression. (A) Cell cycle profile measured by flow cytometry on propidium iodide-stained HeLa cells subjected to RNAi-mediated NM1 gene knockdown or control non-specific RNAi (scrRNAi). Standard ratios of G1, S and G2/M phases are shown in relation to scrRNAi. Where indicated, significances (*p*-values) were calculated by Student's T-test relative to control RNAi. S-phase, *p*_RK605AA_ = 0.0395 (*). (B) Analysis of cell cycle profile on HeLa cells subjected to BDM treatment. The control unsynchronized population of HeLa cells shows standard ratios of G1 and G2/M phases. Increase of the G2/M cell population is observed after a nocodazole block. Prometaphase cells were harvested by mechanical shock and released from the nocodazole block (0 h), in which case the cell cycle profile exhibits only a mitotic population. Two hours after release from the nocodazole block, appearance of the G1 peak indicates that cells have exited mitosis. However, the presence of 20 mM BDM added immediately after release of the nocodazole block led to delayed cell cycle progression with an increased number of cells in mitosis. (C) Cell cycle profiles for HEK293T cells expressing V5-wtNM1, V5-RK605AA NM1, V5-ΔIQ NM1 and V5-ΔC NM1. DNA staining was done by propidium iodide. Where indicated, significances (*p*-values) were calculated by Student's T-test relative to HEK293T cells not expressing any of the V5-tagged NM1 constructs. G1 phase, *p*_ΔC NM1_ = 0.02 (**), *p*_ΔIQ NM1_ = 0.006 (***); S-phase, *p*_RK605AA_ = 0.0008 (***), *p*_ΔC NM1_ = 0.016 (*); G2/M phase, *p*_ΔC NM1_ = 0.007 (***). (D–E) Cell cycle profiles obtained by EdU incorporation for HEK293T cells expressing V5-wtNM1, V5-RK605AA NM1,V5-ΔIQ NM1 and V5-ΔC NM1, following synchronization in G1/S phase with aphidicolin. In Panel (E), the numbers of cells in S phase were quantified 2 h after release from the aphidicolin block.

We next determined whether NM1-mediated transcription activation correlates with cell cycle progression. We expected that it would, since NM1 association with the rRNA gene varies through the cell cycle. We applied flow cytometry to cells subjected to NM1 gene knockdown or to treatment with BDM and measured the distribution through the cell cycle. Remarkably, the number of cells in S phase fell following silencing of the NM1 gene, which indicates a global delay in cell cycle progression (Figure 7A). Likewise, following BDM treatment of synchronized cells, a high percentage of cells were blocked in mitosis 2 h after release from a nocodazole block, when cells should be in telophase/early G1 (Figure 7B). Finally, flow cytometry revealed that stable expression of V5-tagged NM1 mutants also induced alterations in the distribution through the cell cycle. The number of cells in G1 was significantly lower following expression of the V5-wtNM1 and V5-tagged mutants than it was in HEK293T cells, and the number in S-phase fell significantly following the expression of V5-RK605AA NM1 and V5-ΔC NM1 (Figure 7C). Consistently, after 2 h release from an aphidicolin block when cells should be synchronized in early G1/S, analysis of EdU incorporation showed significantly less cells expressing the V5-RK605AA NM1 mutant in S phase compared to wt type (Figure 7D–7E).

Overall our results suggest that impairment of NM1 gene function affects S-phase. Since in cells synchronized in S-phase, H3K9 acetylation levels and PCAF rRNA gene occupancy are significantly increased compared to growing cells (see also Figure S9), we conclude that the marked effect of NM1 gene silencing on the S-phase population is a direct consequence of NM1 down-regulating pol I transcription through an effect on chromatin.

## Discussion

The importance of actin and myosin in gene expression has been convincingly demonstrated [3], [5], [13], but the mechanism through which NM1 facilitates pol I transcription has been a matter of speculation. We have shown here, for the first time, that NM1 plays an important structural role at the rRNA gene promoter that probably connects the pol I machinery with the chromatin. It is possible that NM1 interacts with the polymerase machinery through the pol I-associated actin. Significantly, silencing the NM1 gene and impairment of the myosin motor function both affected the association of actin with rDNA and induced a reduction in rRNA synthesis levels that correlated with increased levels of pol I at the gene promoter. More importantly, stable expression of the V5-RK605AA NM1 mutant, which lacks the ability to bind actin, affected actin occupancy and led to a fall in the rate of rRNA synthesis. We conclude that impairment of the interaction between actin and NM1 induces a transcriptional block that stalls pol I at the gene promoter, thus preventing the activation of transcription. We have shown also that NM1 contacts the rDNA chromatin through its C-terminal tail domain, as has been previously hypothesized [1], [28]. Association with chromatin is not likely to prevent NM1 from interacting with the pol I-associated actin, since the actin-binding domain is located at the N-terminus of the protein. Stable expression of the constructs of NM1 in which the C-terminus has been deleted impaired rRNA synthesis. We conclude that NM1 associates with actin and with the rDNA chromatin to promote the activation of pol I transcription.

We have also shown that NM1 targets the WICH complex through an interaction with SNF2h. This interaction is enhanced when the actin-binding function of NM1 is impaired, suggesting that actin and SNF2h bind to overlapping sites on the NM1 N-terminus and that their associations with the myosin are mutually exclusive. The interaction between NM1 and SNF2h is involved in pol I transcription, as the gene is devoid of NM1 and SNF2h both in mitotic cells, when pol I transcription is temporarily stalled, and in interphase cells treated with low concentrations of actinomycin D, which specifically represses pol I transcription (Figure S10). At the exit of mitosis, the re-associations of NM1 and SNF2h with rDNA are modulated by WSTF. WSTF remains associated with the gene throughout cell division. However, it is probable that mitosis-specific phosphorylation events determine whether WSTF interacts with NM1 and SNF2h at the onset of cell division. We propose that these mechanisms regulate B-WICH disassembly and reassembly at the same time as transcription is activated, presumably by inducing WSTF to switch to distinct chromatin remodeling complexes [22]. When transcription is reactivated, the above mechanisms have a huge impact on the establishment of permissive chromatin. An interesting possibility is that NM1 interacts with SNF2h and stabilizes the B-WICH complex, such that it can subsequently recruit the HAT PCAF. Our results are compatible with a direct interaction of PCAF with the NM1 C-terminus, which if present would be mediated by the IQ motifs. Such an interaction would probably be important for transcription, as is shown by the fact that deletion of the NM1 C-terminus represses rRNA synthesis without interfering with chromatin remodelling. We favour the idea that NM1-mediated PCAF recruitment and H3K9 acetylation occur sequentially and accompany WICH-mediated chromatin remodelling to support active transcription. The different subunits of the B-WICH multiprotein complex may have specific roles that lead to the stepwise modification of chromatin, first through repositioning nucleosomes and then through recruiting HATs for H3K9 acetylation. This mechanism, in turn, ensures that the deposition of UBF is kept under tight control for transcription activation, given that NM1 gene silencing induces an increase in UBF levels on the rDNA unit. UBF levels determine the number of active rRNA genes in mammals [29]. It is tempting to speculate, therefore, that NM1 functions at the gene level as homeostatic regulator of chromatin composition for the activation of pol I transcription and cell cycle progression.

**Figure 8:**
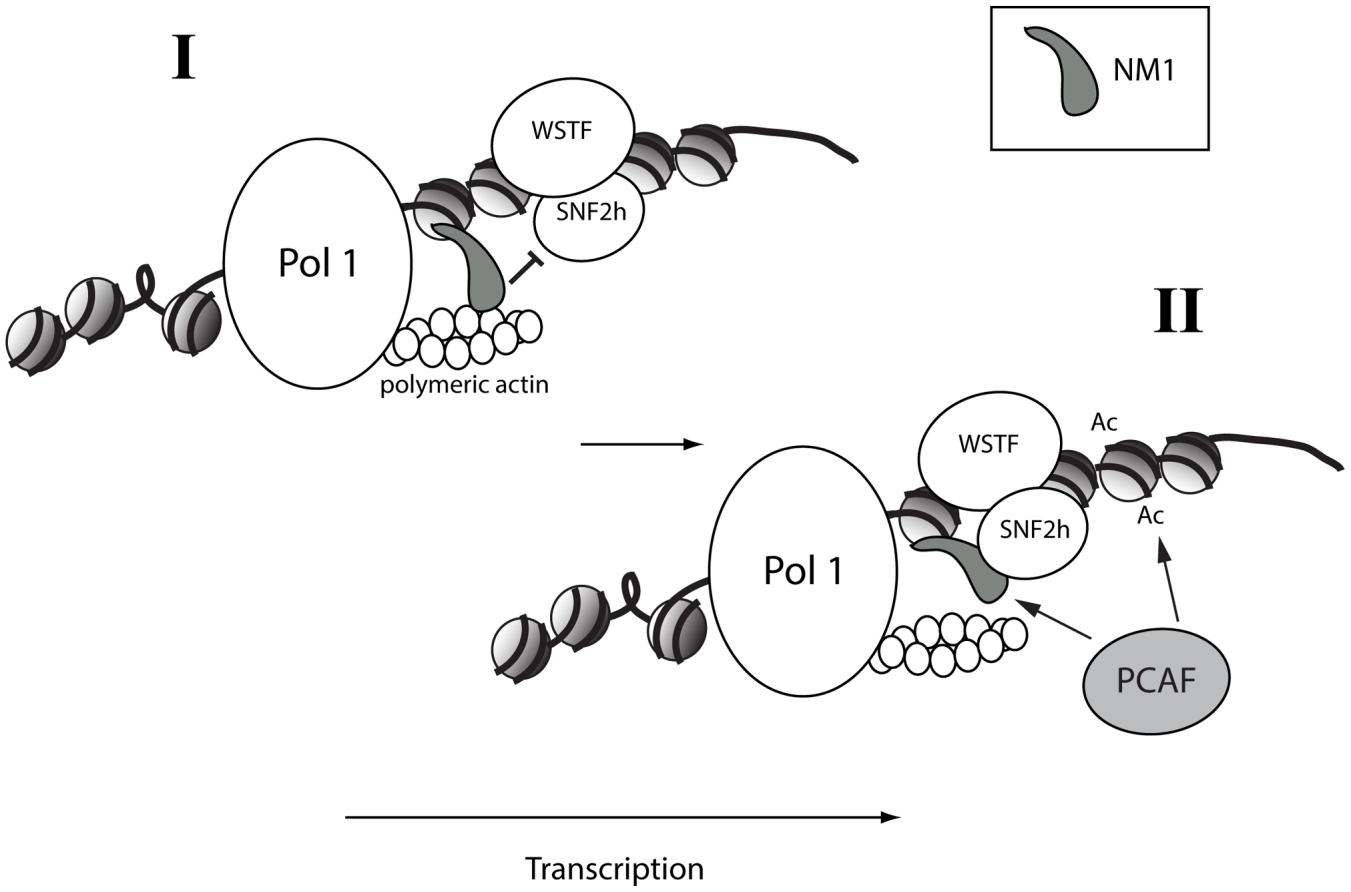
A speculative two-step model in which NM1 bridges the pol I machinery and chromatin *via* an interaction with SNF2h that competes with actin. (I) NM1 interacts with polymeric actin and with the rDNA *via* its C-terminus, generating local force that pulls the polymerase along active gene. (II) Upon NM1 dissociation from actin, NM1 interacts with SNF2h in a WSTF-dependent manner, a mechanism that provides a way to stabilize the WICH complex on the rDNA, to recruit PCAF, and to maintain the levels of H3K9 acetylation required for transcription activation.

One of the key questions is why the binding of actin and SNF2h to NM1 are mutually exclusive. Members of the myosin 1 family have short tails and are low-duty-ratio motors with a low affinity for actin [30]–[34]. This means that NM1 is not likely to support cargo movements over long distances, but may be involved mainly in defining the structure and organization of the elongating pol I machinery with respect to its chromatin template. The importance of the actin-binding and ATPase activities of NM1 for pol I transcription suggest that it is necessary to create force locally. Polymeric actin interacts with pol I, an interaction that is required for transcription [8]. Actin polymerization is tightly regulated along active genes by cofilin 1, a protein that severs F-actin [8], [9]. Therefore throughout the myosin cycle [35], [36], concomitantly with the establishment of an actomyosin complex, force generation may primarily result from the direct interaction of NM1 with rDNA through its C-terminus and the simultaneous pulling of polymeric actin attached to the polymerase (Figure 8, Panel I). When NM1 does not interact with actin, a condition mimicked by the V5 RK605AA NM1 mutant (Figure S5A–S5B), the rDNA associated-NM1 interacts directly with SNF2h in a manner that requires WSTF. This provides a mechanism to stabilize the chromatin remodelling complex at active gene promoters when pol I transcription is activated, in late mitotic and interphase cells (Figure 8, Panel II). Stable expression of the V5-tagged RK605AA NM1 mutant induced the establishment of a more compact chromatin state at the rRNA gene promoter, which is consistent with the repression of transcription. We propose that the two-step mechanism is based on the NM1-actin interaction; it facilitates polymerase tread-milling along active genes to provide permissive chromatin for transcription elongation by modulating B-WICH assembly and PCAF recruitment. At the exit of mitosis, these mechanisms probably have a huge impact on cellular growth and proliferation when there is a high demand for protein synthesis and when a fraction of rDNA must be kept in an active configuration.

## Methods

### Antibodies and reagents

The antibodies against WSTF (ab50850), SNF2h (ab3749) and H3 (ab1791) were from Abcam. The antibodies against PCAF (sc13124) and UBF (sc9131) were purchased from SantaCruz. The antibodies to actin are specific for the β-isoform and were purchased from Sigma Aldrich (clone AC74) and from Abcam (ab8226). The antibody against the V5 epitope (A190-120A) was purchased from Bethyl Laboratories. The non-specific rabbit IgGs (ab46540) were from Abcam. The anti-pol I antibody was a kind gift of T. Moss (McGill University, Canada) and the antibody against NM1 has previously been characterized [6]. For immunofluorescence the rabbit polyclonal anti-WSTF antibody (2152) was purchased from Cell Signalling, the mouse monoclonal antibody against fibrillarin (ab4566) was from Abcam and the human autoimmune sera S57299 against pol I (specific for RPA194) and UBF were gifts from U. Scheer (University of Wurzburg, Germany) [10] and D. Hernandez-Verdun (Institut Monod, Paris, France) [37]. The H3K9Ac (ab10812) and H3K14Ac (ab52946) antibodies were purchased from Abcam. The monoclonal antibody to bromouridine triphosphate (BrdU) to monitor FUrd incorporation was from Sigma-Aldrich. Species-specific secondary antibodies conjugated to Cy2, Alexa 488, Alexa 568, Alexa 594 or Texas Red were from Invitrogen and Jackson ImmunoResearch. DNA was revealed by DAPI staining (300 nM for 3 min at room temperature, RT).

### Cell culture and synchronization

HeLa cells were grown in DMEM medium (Gibco), supplemented with 10% foetal bovine serum (Gibco) and a 1% penicillin/streptomycin cocktail (Gibco). For nocodazole synchronization, cells were treated with nocodazole (Sigma-Aldrich) added to a final concentration of 40 ng/ml (0.133 µM) for 4 h or 16 h at 37°C. Mitotic cells were harvested by mechanical shock. Nocodazole release was done by suspension sedimentation in fresh DMEM medium. For synchronization with aphidicolin (Sigma-Aldrich), cells were blocked in G1/S phase by incubating them with aphidicolin at a final concentration of 2 µg/ml for 16 h at 37°C. For statistical analysis and immunofluorescence cells were seeded on poly-L-lysine coated coverslips. For ChIP analysis, cells were seeded on 175 cm^2^ culture dishes. Where indicated, cells were incubated with BDM to a final concentration of 20 mM for 2 h at 37°C as previously described [6]. HEK293T cells and HEK293T cells constitutively expressing V5-tagged wtNM1 and RK605AA NM1 point mutants, as well as ΔIQ NM1 and ΔC NM1 deletion mutants were kind gifts of I. Grummt (University of Heidelberg, Germany).

### Transcription assays, NM1 RNAi, and immunofluorescence

To reveal active pol I transcription living cells grown on cover slips were incubated with 2 mM FUrd (Sigma-Aldrich) in DMEM and incubation was allowed for 5–8 min [25], [26]. For detection of incorporated FUrd, cells were fixed with a 3.7% formaldehyde solution in PBS at room temperature, permeabilized with a 0.5% Triton X-100 solution in PBS and incubated with the indicated antibodies. Where indicated, FUrd incorporation was performed on HeLa cells subjected to RNAi-mediated NM1 gene knockdown essentially as described in [7]. Duplexes against NM1 or control scrambled versions [7] were applied by transfection with Lipofectamine RNAi Max (Invitrogen) at a final concentration of 30 nM. Total RNA was extracted with the TRI reagent as specified by the manufacturer (Sigma). For analysis of nascent pre-rRNA, total RNA isolated from NM1-silenced or control cells, and from HEK293T stably expressing V5-wtNM1, V5-RK605AA NM1, V5-ΔC NM1 or V5-ΔIQ NM1 was analyzed by qRT-PCR with specific primers amplifying 45S pre-rRNA (forward, 5′ GGT ATA TCT TTC GCT CCG AG; reverse, 5′ AGC GAC AGG TCG CCA GAG GA) and GAPDH mRNA (forward, 5′ GCA TCC TGC ACC ACC AAC TC; reverse, 5′ ACG CCA CAG CTT TCC ACA GG). qRT-PCR was performed using SYBR-green from Applied Biosystems according to the manufacturer's instructions (see also below for further details). For the FUrd incorporation on NM1-silenced cells or on cells treated with BDM, quantification of the fluorescence signal intensities was done on images obtained from wide-field imaging as described by Ordlik et al. [38].

Wide-field imaging was also used [38] to collect examples of mitotic cells immunolabelled with anti-WSTF, SNF2h and NM1 antibodies and to establish the statistical analyses of the mitotic stages at the DAPI level. Otherwise images were obtained from a confocal microscope (Zeiss LSM meta) with 63× oil objective NA 1.3. Images were collected and analyzed using the LSM software and Photoshop.

### Flow cytometry

Control HeLa cells, cells transfected with control scrambled siRNA oligonucleotides or siRNA oligonucleotides targeting the NM1 gene [7] and HEK293T expressing V5-tagged NM1 constructs were analyzed by flow cytometry (FACS) according to standard protocols. Briefly, cells were trypsinized, washed in PBS by suspension/sedimentation and fixed in 70% ethanol. Following 15 min incubation on ice, cells were pelleted at 1500 rpm for 5 min. DNA was labelled specifically by incubating cell pellets with a PI/RNase A solution (50 µg/ml PI, 0.1 mg/ml RNaseA, 0.05% Trition X-100 in PBS) for 40 min at 37°C. The DNA content was measured by FACS with a FACSCalibur (Becton Dickinson). Every experiment was repeated at least three times. A total of 10,000 events were counted in all cases. Cell cycle phases were analyzed using the FlowJo software and depicted as percentages. Where indicated, *p*-values were calculated by an unpaired two-tailed Student's T-test. For FACS analysis of HEK293T cells expressing V5-tagged NM1 constructs synchronized with aphidicolin, either at t = 0 or 2, 4 or 6 h after release from the block, cells were treated with 10 µM Click-iT EdU Alexa Fluor 647 flow cytometry assay kit for 10 minutes (Invitrogen). Briefly, after harvesting, cells were fixed and permeablized with 1× saponin, stained with an Alexa flour 647 cocktail as described in the manufacturer's instruction manual and subsequently stained with a PI cocktail (50 µg/ml PI, 0.1 mg/ml RNaseA). DNA content was measured with FACSCalibur (Becton Dickinson). Cells were analyzed using CellQuest Pro software.

### Phosphorylation assays

Phosphorylation assays were performed essentially as described [22]. Lysates were prepared from growing HeLa cells, from HeLa cells blocked in prometaphase or from HeLa cells 2 h after release from the block. Briefly, lysis was carried out in 0.7 M KCl, 20 mM Tris at pH 8.0, 0.1 mM EDTA, 2 mM PMSF, 0.4% NP40, 10% glycerol and PhosSTOP (Roche). The lysate was separated by 7% SDS-PAGE containing up to 100 µmol Phos-tag™ AAL-107 according to the manufacturer's instructions (MANAC Incorporated) and 20 µmol MnCl_2_, and transferred to a PVDF membrane using a transfer buffer containing 48 mM Tris, 39 mM glycine, 1.3 mM SDS, 5% methanol and 25 µM EDTA. Detection was done with a rabbit polyclonal antibody against WSTF and actin. Where indicated mitotic extracts were subjected to phosphatase treatment as described in the instruction manual provided by the manufacturer (New England Biolabs).

### Protein–protein interaction assays and WSTF gene silencing

Co-immunoprecipitations of endogenous NM1, WSTF and SNF2h from mitotic or growing HeLa cells were done following standard procedures [12]. Where indicated immunoprecipitations were performed on lysates obtained from growing HeLa cells subjected to WSTF gene knockdown as described in [10], [12]. For immunoprecipitations of constitutively expressed V5-tagged wtNM1, RK605AA NM1, ΔIQ NM1 and ΔC NM1 mutants from HEK293T cells, total cell lysates were incubated with the anti-V5 antibody. The antibodies were subsequently precipitated with Protein G Sepharose (Invitrogen). Precipitated proteins were washed with 20 volumes of RIPA buffer (containing 1XPBS, 1 mM PMSF, 0.2% NP-40, 0.1% deoxycholate, 0.1% SDS). The beads were resuspended in Laemmli buffer and heat denatured. Bound proteins were resolved by SDS-PAGE and analyzed on immunoblots for V5 epitope, SNF2h, PCAF or actin.

### High-resolution MNase assay

These experiments were essentially performed as described by Petesch and Lis, 2008 [27]. Briefly, HeLa cells subjected to NM1 gene knockdown, HeLa cells treated with control scrRNAi oligonucleotides (see above) as well as HEK293T stably expressing V5-wtNM1, V5-RK605AA NM1 or V5-ΔC NM1 were cross-linked with 1% formaldehyde for 20 minutes. In each case, the chromatin was prepared as for ChIP (see below), but washed with Buffer D containing 25% glycerol, 5 mM magnesium acetate, 50 mM Tris at pH 8.0, 0.1 mM EDTA, 5 mM DTT. Before enzymatic digestion the chromatin was sonicated lightly in MNase buffer (60 mM KCl, 15 mM NaCl, 15 mM Tris at pH 7.4, 0.5 mM DTT, 0.25 M sucrose, 1.0 mM CaCl2), 8 times for 30 seconds. The equivalent of 0.46×10^6^ cells was used in each reaction, and the level of DNA was first adjusted to be in the same range in the samples from all different treatments. The amount of MNase was optimized for each experiment, with several MNase concentrations used such that the reaction occurred in the linear range of digestion. Two samples from each treatment were used for the calculations: 0 U MNase and one concentration between 10 U and 20 U MNase. The reactions were performed at 37°C for 30 min and then stopped by adding 12.5 mM EDTA/0.5% SDS. After 3 h proteinase K treatment, the cross-linking was reversed at 65°C for 5 h. DNA was extracted [11] and the digest was evaluated by qPCR using the primer pairs shown in Table S2, giving a product of approximately 100 bp. The results were analyzed by calculating ΔCt between the reactions performed with and without MNase. The values are presented as 2ΔCt. Chromatin from cells transfected with control siRNA oligonucleotides and chromatin from untransfected gave the same MNase digestion pattern.

### Chromatin immunoprecipitation and qPCR analysis

ChIP on growing or synchronized HeLa cells was performed as previously described [12]. Briefly, formaldehyde cross-linked chromatin was obtained from HeLa cells in interphase, prometaphase, early G1 treated or early G1 untreated with BDM (20 mM) and S-phase, as previously described [6]. In all cases cross-linked chromatin was immunoprecipitated with antibodies to pol I, UBF, WSTF, SNF2h, NM1, H3, H3K14Ac, H3K9Ac, PCAF and non-specific rabbit IgGs. DNA-protein complexes were analyzed by qPCR with specific primers amplifying multiple regions of the rRNA gene, including promoter, ETSs, ITSs, 18S, 28S and IGS (see Table S1 for sequences). qPCR was performed using SYBR-green from Applied Biosystems according to the manufacturer's instructions. The primer concentration was 2.5 mM and the samples analyzed by Rotor-Gene 6000 series software 1.7. The PCR conditions were: hold 95°C for 3 minutes, followed by cycles of 95°C for 3 seconds, 60°C for 20 seconds, 72°C for 3 seconds. The results were analyzed using an average of Ct of no antibody and IgG as background. The 2^ΔCt^ of each sample in triplicates was related to the 2^ΔCt^ of the input sample.

ChIP assays were also performed on formaldehyde crosslinked chromatin isolated from wt HEK293T and HEK293T cells expressing V5-tagged wtNM1, RK605AA NM1, ΔIQ NM1 and ΔC NM1 mutants using antibodies against the V5 epitope, WSTF, SNF2h, actin and PCAF as well as non-specific rabbit IgGs. Precipitated chromatin was analyzed by qPCR with the same primers as in Table S1.

## Supporting Information

Figure S1 Monitoring exit from mitosis after cell synchronization. Examples of wide-field images showing mitotic HeLa cells at t = 0 min and early G1 HeLa cells at t = 120 min from the nocodazole block release (scale bar 10 µm). DAPI staining was performed to visualize the DNA and to identify the mitotic stages by morphology.
(TIF)

Figure S2 Analysis of pol I transcription in living cells. (A) Pol I transcription in unsynchronized cells analyzed by double immunostaining for incorporated FUrd (red) with UBF or pol I (green) as indicated in interphase and mitotic cells (scale bars 5 µm). (B) Pol I transcription reactivation in synchronized cells. FUrd was added to the medium at the same time as with the release from nocodazole block. Incorporated FUrd (red) co-localises with UBF (green) on pol I transcription foci in telophase and early G1 cells (scale bars 5 µm). Insets are twofold enlargements of the co-localization foci indicated by white arrows.
(TIF)

Figure S3 In living cells, WSTF, SNF2h and NM1 transit through actively transcribing NORs. Cells were synchronized in prophase and subjected to a short FUrd pulse just before reaching telophase or early G1. Double immunostaining was performed with antibodies to (A) FUrd and NM1, (B) FUrd and WSTF and (C) FUrd and SNF2h. Specimens were imaged by confocal microscopy (scale bar 5 µm).
(TIF)

Figure S4 Distributions of NM1, SNF2h and WSTF in early mitotic HeLa cells. Localization was monitored by immunostaining (green) with the indicated antibodies and DAPI for DNA detection. Analysis was by confocal microscopy (scale bar 5 µm).
(TIF)

Figure S5 (A) Schematic representation of V5-tagged wt and mutated NM1 constructs stably expressed in HEK293T cell lines and used in the study. (B) Co-precipitations of actin from total lysates obtained from HEK293T cells constitutively expressing wt and mutated V5-tagged NM1 constructs using anti-V5 antibodies. Bound proteins are detected on immunoblots for V5 and actin. 5% of the input is shown in Lane 1. IP, immunoprecipitation. (C) Schematic representation of the human rDNA transcription unit and positions of PCR products. (D) ChIP assays performed on transfected HEK293T cells expressing V5-tagged wt NM1 or mutated NM1 constructs (as indicated) and HEK293T cells not expressing any of the NM1 constructs with antibodies against V5, histone H3 and non-specific rabbit immunoglobulins (IgG). Co-precipitated DNA was analyzed by PCR with primers for rDNA promoter, 18S rDNA and 28S rDNA. PCR products were resolved by 2% agarose gel electrophoresis (and revealed by ethidium bromide staining as seen above).
(TIF)

Figure S6 rRNA gene occupancy of the HAT PCAF requires a functional NM1. (A) Schematic representation of the rDNA transcription unit. (B–C) ChIP assays on chromatin from HEK293T cells and HEK293T cells stably expressing V5-wtNM1, V5-RK605AA NM1, V5-ΔC NM1 and V5-ΔIQ NM1 mutants with antibodies against V5 and PCAF as indicated below the x-axis. qPCR analysis was performed with primers amplifying (B) promoter and (C) 18S rDNA. The values are presented as the percentage of the input signal for each pair. Error bars represent standard deviations.
(TIF)

Figure S7 FUrd incorporation assays performed on (A) HeLa cells subjected to NM1 gene silencing or control experiments with RNAi oligonucleotides with scrambled sequences (scrRNAi), and (B) HeLa cells incubated with BDM or DMSO. In all cases, detection of incorporated FUrd was with a fluorochrome-conjugated anti-BrdU antibody. Detection was by confocal microscopy. Scale bars, 10 µm.
(TIF)

Figure S8 (A) Schematic representation of V5-tagged wt and mutated NM1 constructs stably expressed in HEK293T cell lines and used in the study. (B) Chromatin profile from HEK293T cells stably expressing V5-wtNM1, V5-RK605AA NM1 and V5-ΔC NM1 compared to V5-ΔIQ NM1 shown as 2ΔCt of undigested and MNase digested cross-linked chromatin. The position of each primer pair is indicated below the graph; 2c (coding), Position 2 in the coding region. Error bars represent standard deviations of three separate experiments.
(TIF)

Figure S9 PCAF occupancy and H3K9 acetylation patterns on rRNA genes analyzed in growing HeLa cells and in cells arrested in S-phase. (A) FACS analysis on growing HeLa cells, HeLa cells arrested in G1/S with aphidicolin, HeLa cells in S-phase 4 h after release from the aphidicolin block and in S/G2 phase 6 h after release from the aphidicolin block. (B) ChIP analysis on chromatin isolated from growing HeLa cells and cells in S-phase 4 h after release from the aphidicolin block, using antibodies to PCAF and acetylated H3K9, as indicated below the x-axis. qPCR analysis was performed with primers amplifying rRNA gene promoter. The values are presented as the percentage of the input signal for each pair. Error bars represent standard deviations. *p*_PCAF_ = 0.00378 (**), *p*_RK605AA_ = 0.00018 (***).
(TIF)

Figure S10 (A) Schematic representation of the human rDNA transcription unit and positions of PCR products. (B) ChIP on HeLa cells subjected to a pol I transcriptional block with actinomycin D (50 ng/ml) using the antibodies indicated below the bars. The values are presented as the percentages of the input signal for each primer pair. Error bars represent standard deviations from four separate experiments.
(TIF)

Table S1 Sequences of primers used in the qPCR analyses of ChIP experiments.
(DOC)

Table S2 Sequences of primers used in the MNase experiment [4].
(DOC)
