# Supplementary figures and images for: Nuclear Myosin 1c Facilitates the Chromatin Modifications Required to Activate rRNA Gene Transcription and Cell Cycle Progression
Source: PLoS Genet. 2013 Mar 21;9(3):e1003397. doi: 10.1371/journal.pgen.1003397 (PMC3605103; doi:10.1371/journal.pgen.1003397)

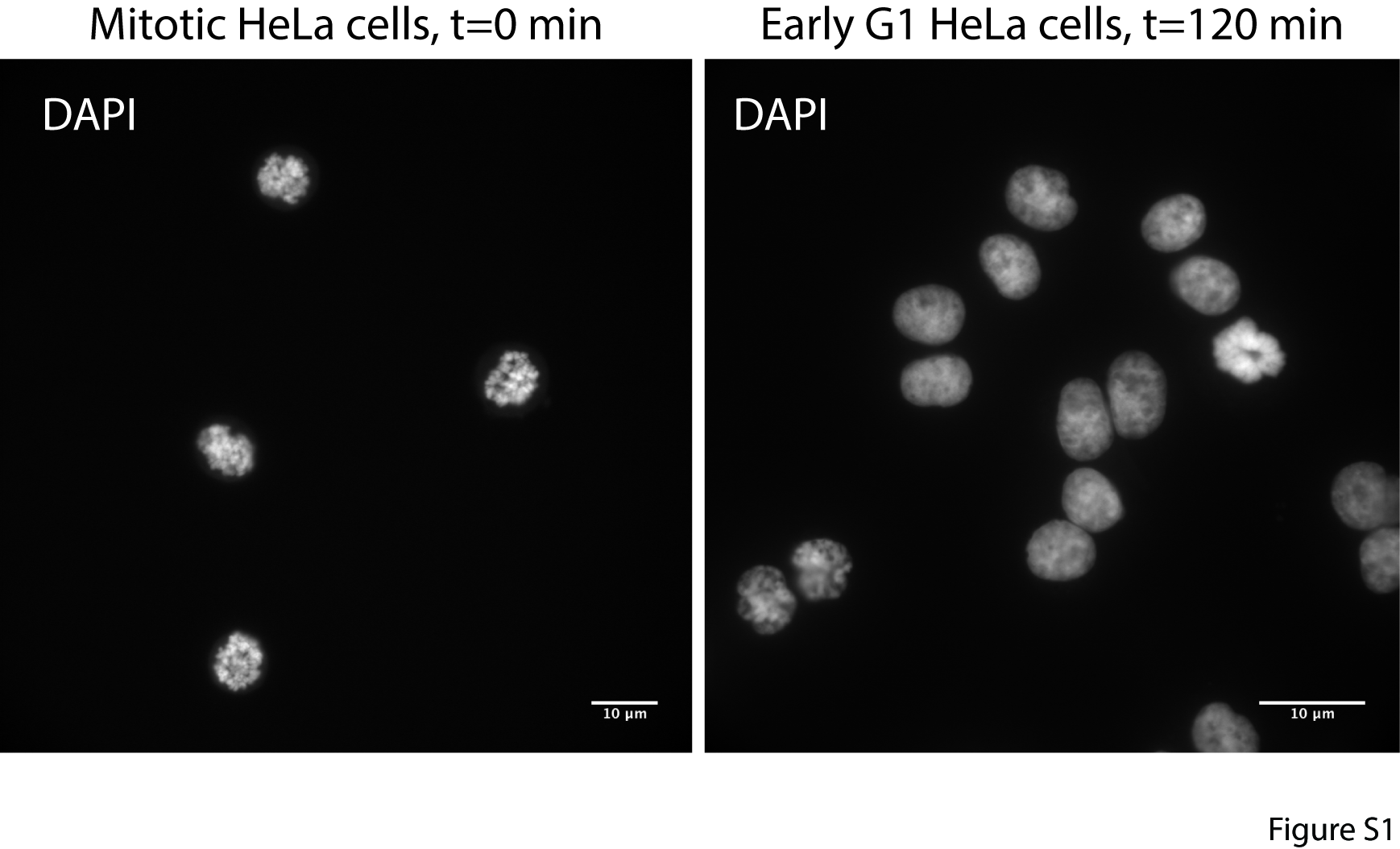

Supplement: Figure S1 — Monitoring exit from mitosis after cell synchronization. Examples of wide-field images showing mitotic HeLa cells at t = 0 min and early G1 HeLa cells at t = 120 min from the nocodazole block release (scale bar 10 µm). DAPI staining was performed to visualize the DNA and to identify the mitotic stages by morphology. (TIF) [file pgen.1003397.s001.tif]

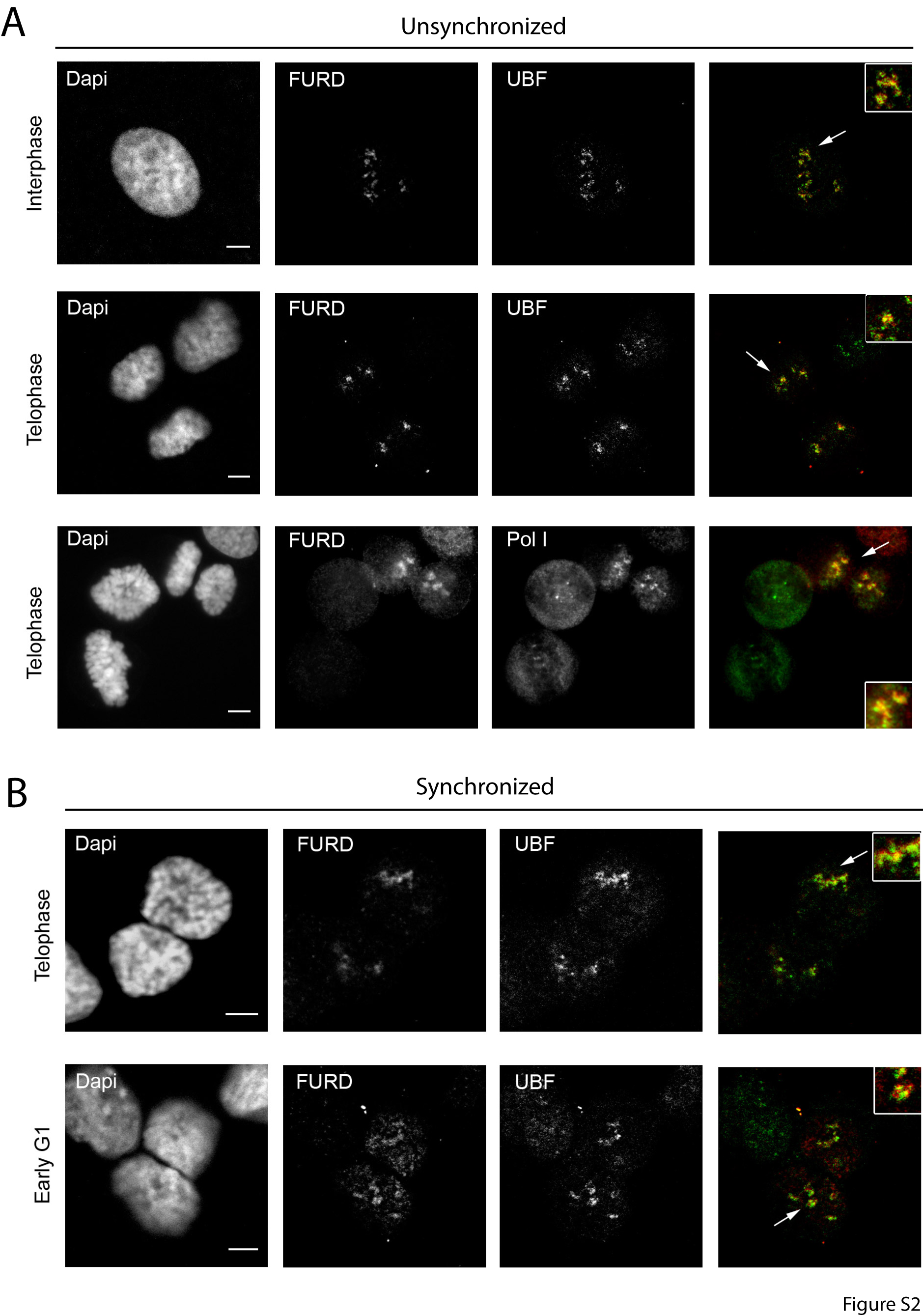

Supplement: Figure S2 — Analysis of pol I transcription in living cells. (A) Pol I transcription in unsynchronized cells analyzed by double immunostaining for incorporated FUrd (red) with UBF or pol I (green) as indicated in interphase and mitotic cells (scale bars 5 µm). (B) Pol I transcription reactivation in synchronized cells. FUrd was added to the medium at the same time as with the release from nocodazole block. Incorporated FUrd (red) co-localises with UBF (green) on pol I transcription foci in telophase and early G1 cells (scale bars 5 µm). Insets are twofold enlargements of the co-localization foci indicated by white arrows. (TIF) [file pgen.1003397.s002.tif]

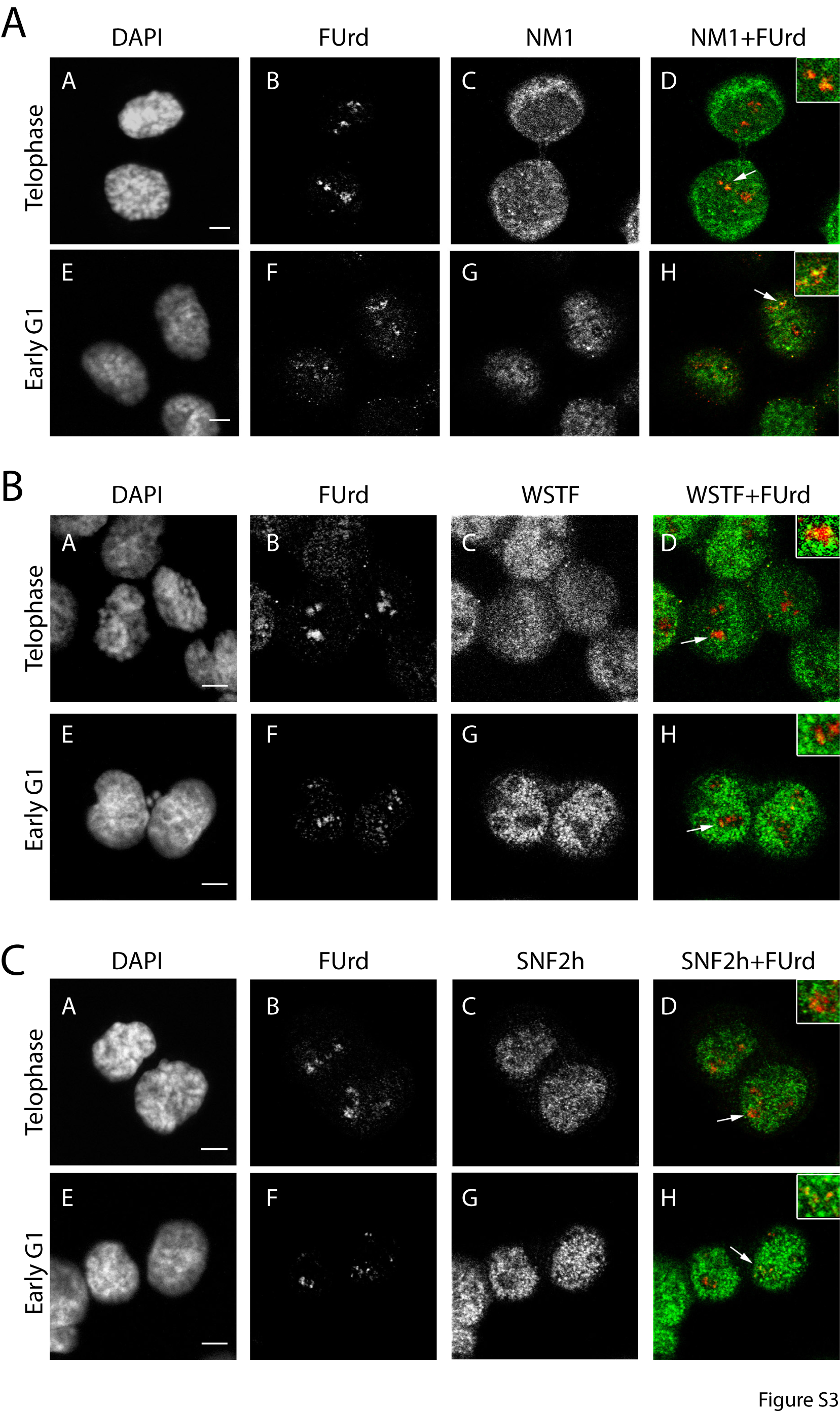

Supplement: Figure S3 — In living cells, WSTF, SNF2h and NM1 transit through actively transcribing NORs. Cells were synchronized in prophase and subjected to a short FUrd pulse just before reaching telophase or early G1. Double immunostaining was performed with antibodies to (A) FUrd and NM1, (B) FUrd and WSTF and (C) FUrd and SNF2h. Specimens were imaged by confocal microscopy (scale bar 5 µm). (TIF) [file pgen.1003397.s003.tif]

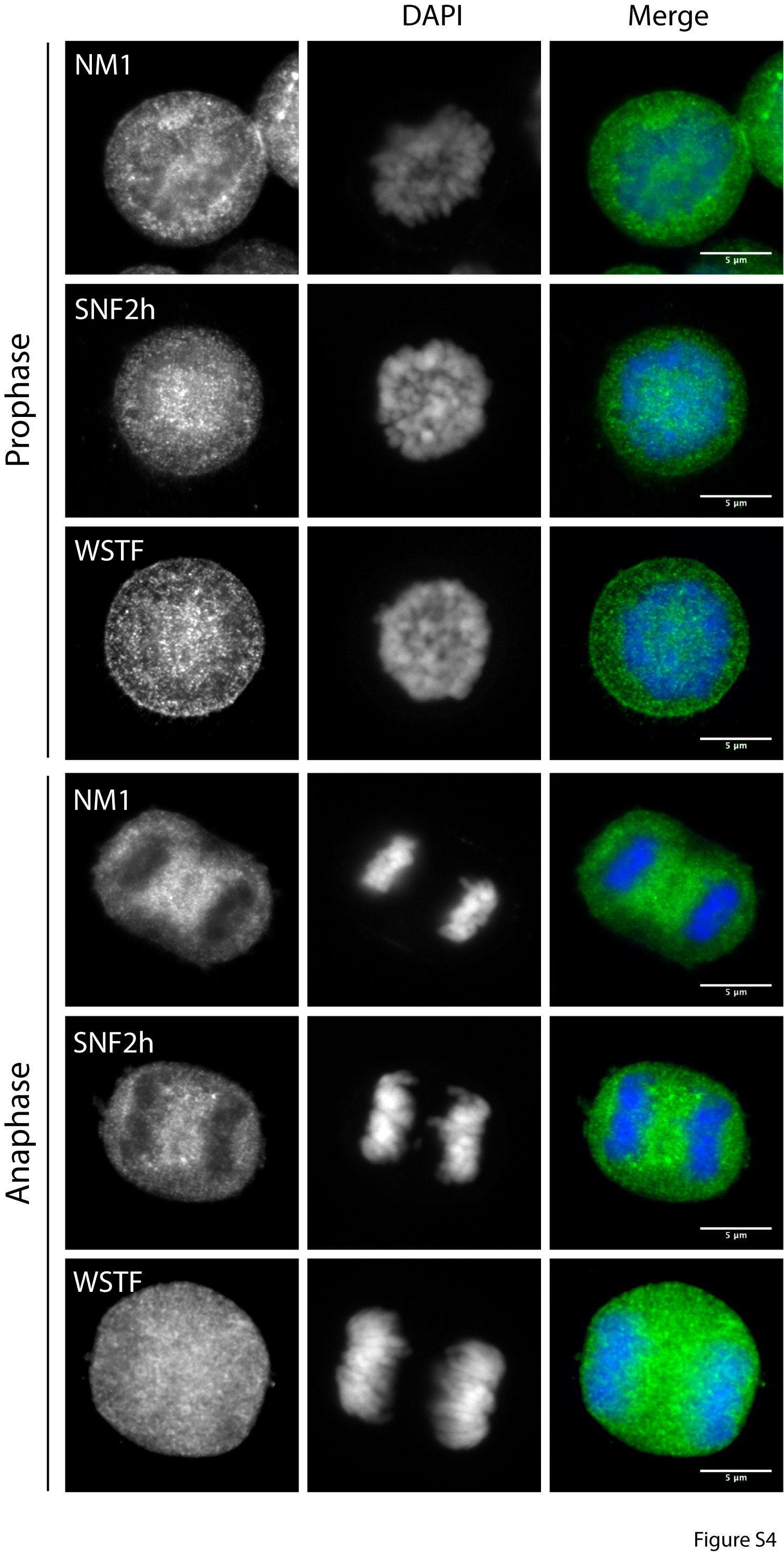

Supplement: Figure S4 — Distributions of NM1, SNF2h and WSTF in early mitotic HeLa cells. Localization was monitored by immunostaining (green) with the indicated antibodies and DAPI for DNA detection. Analysis was by confocal microscopy (scale bar 5 µm). (TIF) [file pgen.1003397.s004.tif]

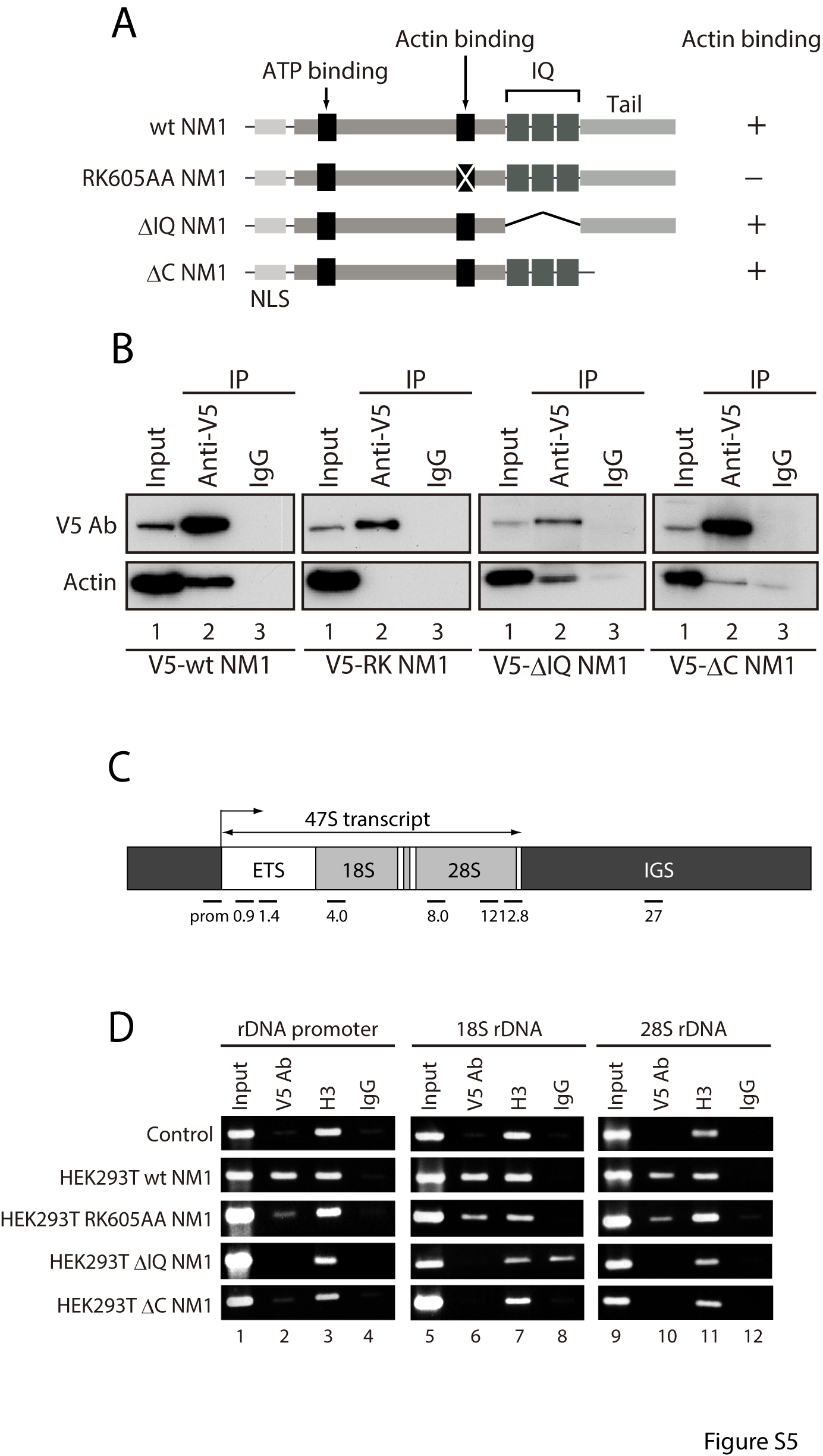

Supplement: Figure S5 — (A) Schematic representation of V5-tagged wt and mutated NM1 constructs stably expressed in HEK293T cell lines and used in the study. (B) Co-precipitations of actin from total lysates obtained from HEK293T cells constitutively expressing wt and mutated V5-tagged NM1 constructs using anti-V5 antibodies. Bound proteins are detected on immunoblots for V5 and actin. 5% of the input is shown in Lane 1. IP, immunoprecipitation. (C) Schematic representation of the human rDNA transcription unit and positions of PCR products. (D) ChIP assays performed on transfected HEK293T cells expressing V5-tagged wt NM1 or mutated NM1 constructs (as indicated) and HEK293T cells not expressing any of the NM1 constructs with antibodies against V5, histone H3 and non-specific rabbit immunoglobulins (IgG). Co-precipitated DNA was analyzed by PCR with primers for rDNA promoter, 18S rDNA and 28S rDNA. PCR products were resolved by 2% agarose gel electrophoresis (and revealed by ethidium bromide staining as seen above). (TIF) [file pgen.1003397.s005.tif]

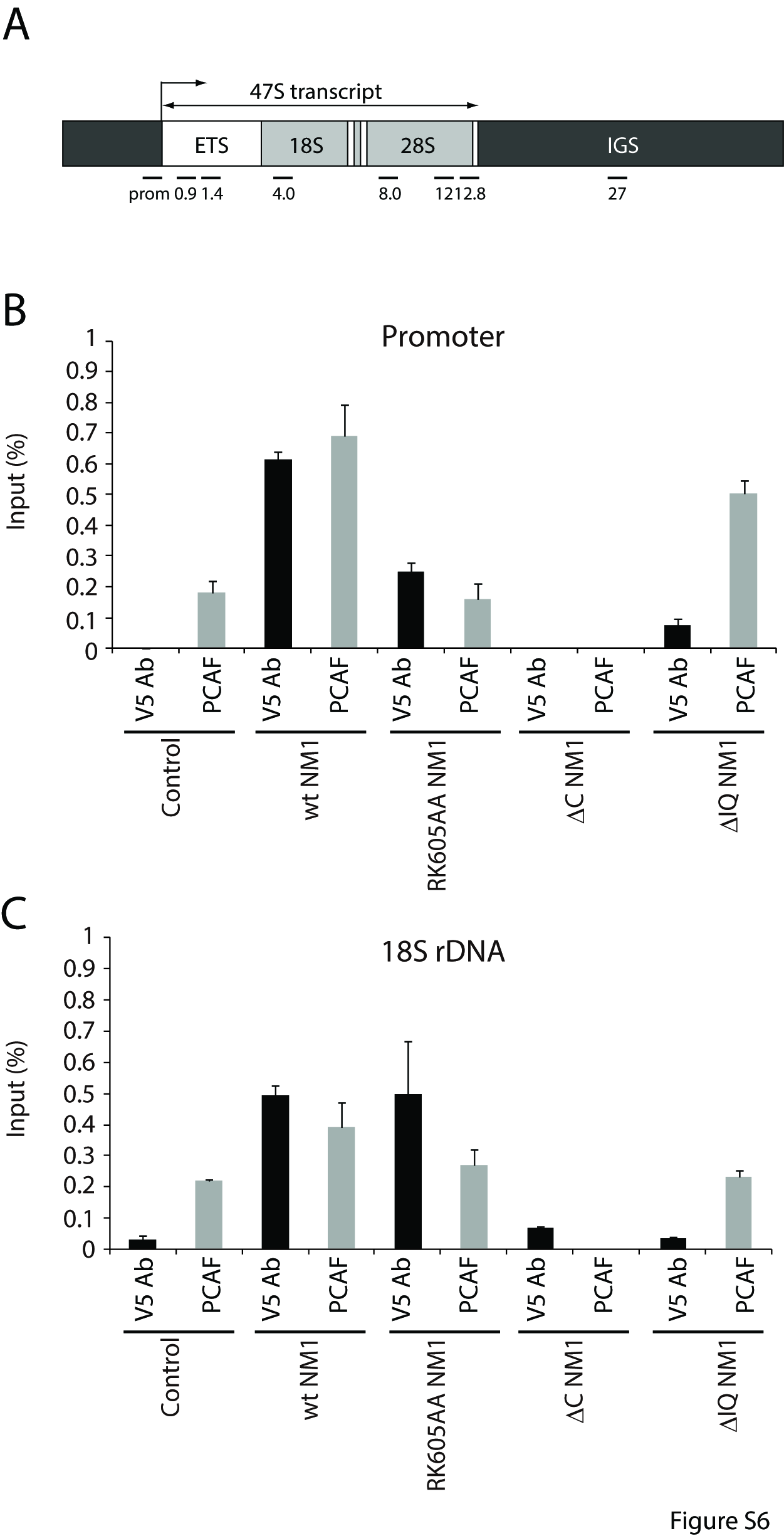

Supplement: Figure S6 — rRNA gene occupancy of the HAT PCAF requires a functional NM1. (A) Schematic representation of the rDNA transcription unit. (B–C) ChIP assays on chromatin from HEK293T cells and HEK293T cells stably expressing V5-wtNM1, V5-RK605AA NM1, V5-ΔC NM1 and V5-ΔIQ NM1 mutants with antibodies against V5 and PCAF as indicated below the x-axis. qPCR analysis was performed with primers amplifying (B) promoter and (C) 18S rDNA. The values are presented as the percentage of the input signal for each pair. Error bars represent standard deviations. (TIF) [file pgen.1003397.s006.tif]

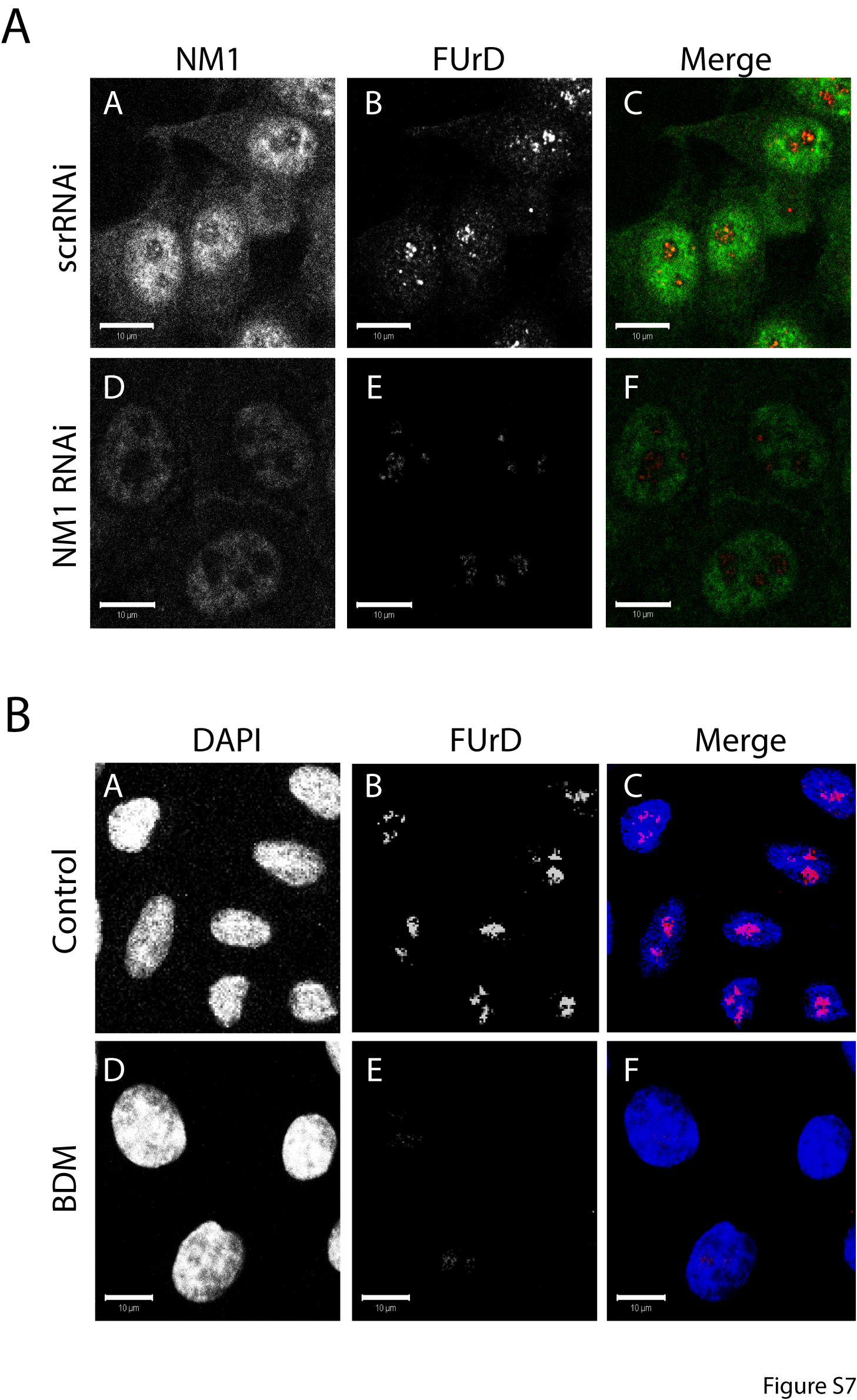

Supplement: Figure S7 — FUrd incorporation assays performed on (A) HeLa cells subjected to NM1 gene silencing or control experiments with RNAi oligonucleotides with scrambled sequences (scrRNAi), and (B) HeLa cells incubated with BDM or DMSO. In all cases, detection of incorporated FUrd was with a fluorochrome-conjugated anti-BrdU antibody. Detection was by confocal microscopy. Scale bars, 10 µm. (TIF) [file pgen.1003397.s007.tif]

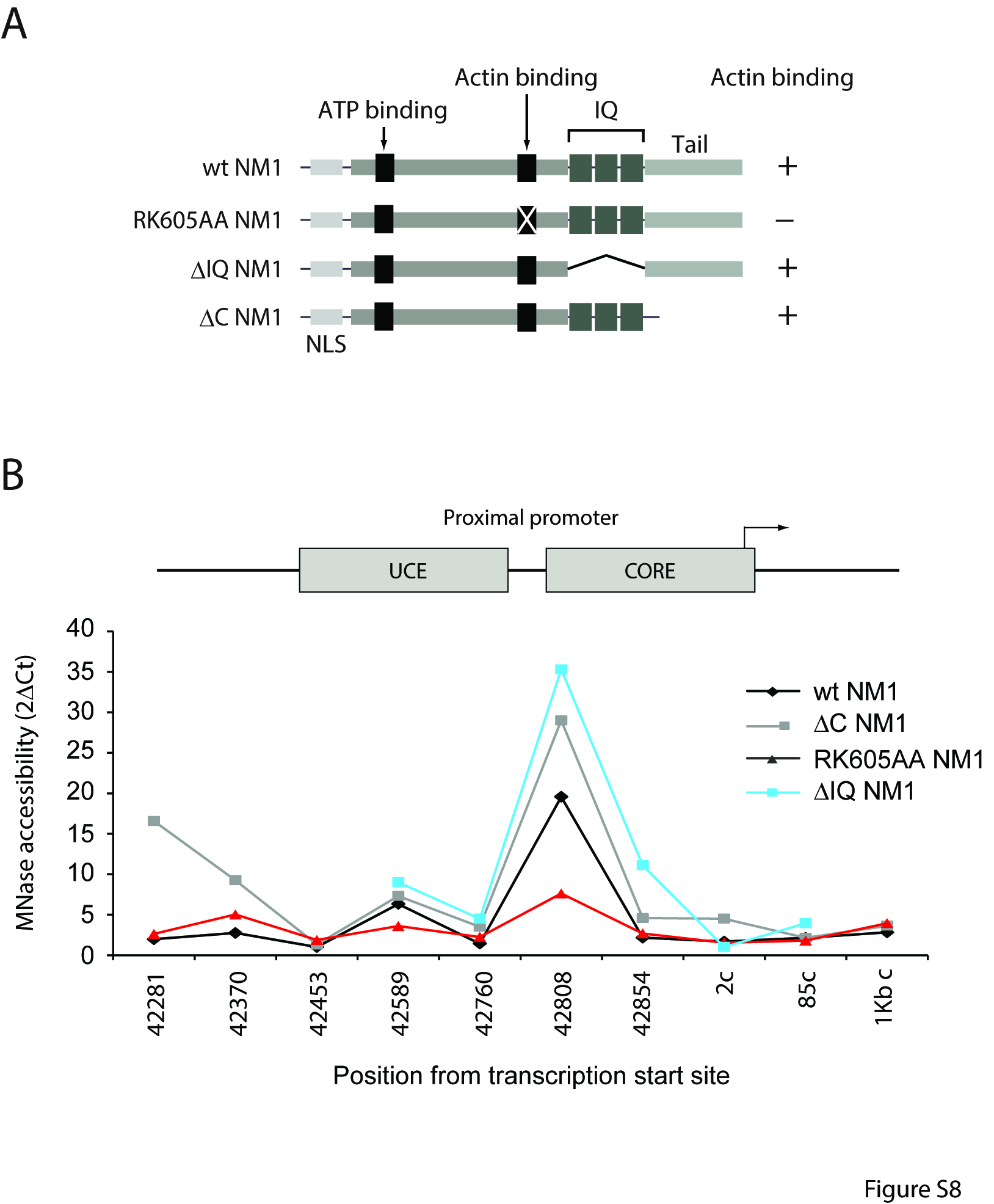

Supplement: Figure S8 — (A) Schematic representation of V5-tagged wt and mutated NM1 constructs stably expressed in HEK293T cell lines and used in the study. (B) Chromatin profile from HEK293T cells stably expressing V5-wtNM1, V5-RK605AA NM1 and V5-ΔC NM1 compared to V5-ΔIQ NM1 shown as 2ΔCt of undigested and MNase digested cross-linked chromatin. The position of each primer pair is indicated below the graph; 2c (coding), Position 2 in the coding region. Error bars represent standard deviations of three separate experiments. (TIF) [file pgen.1003397.s008.tif]

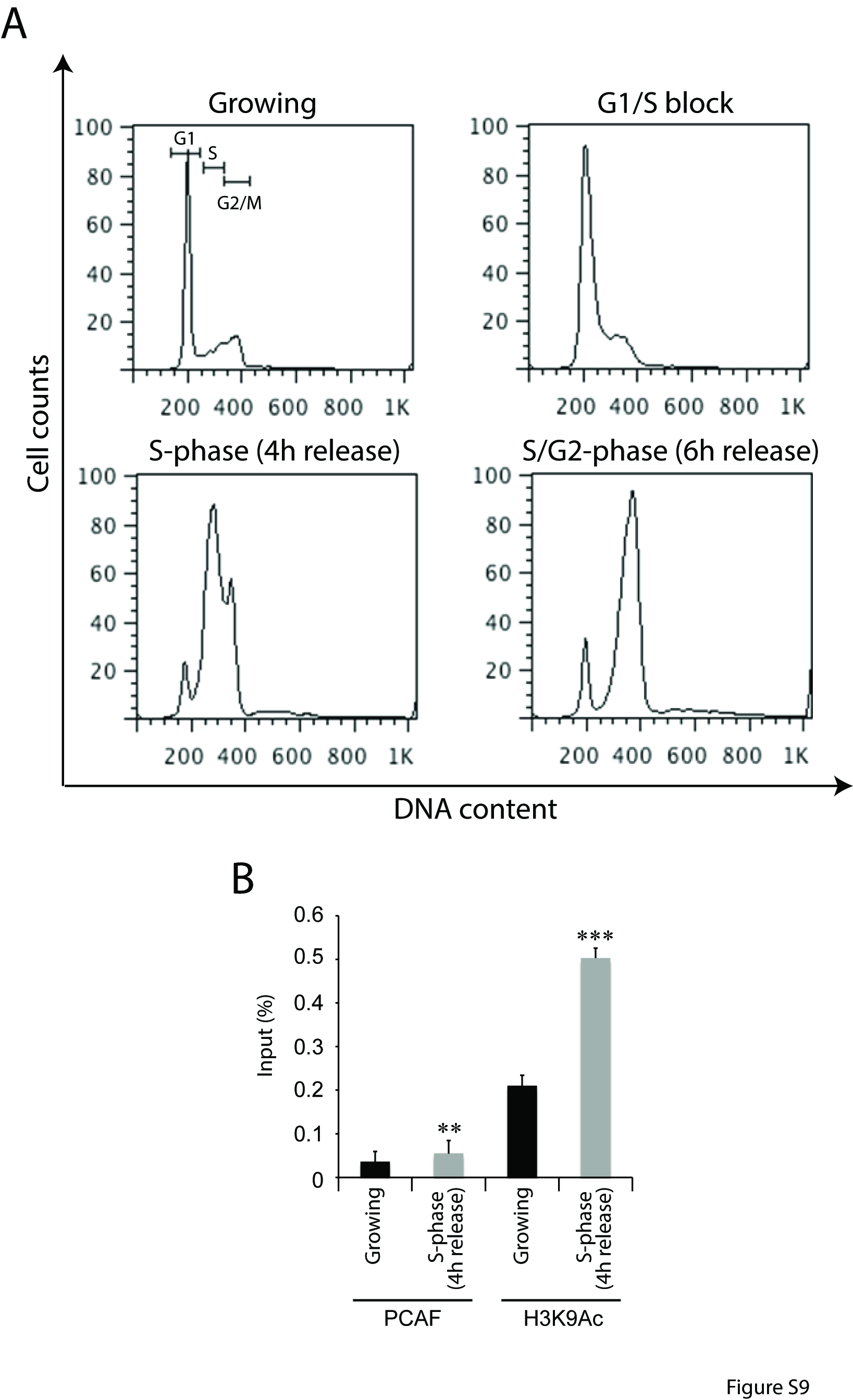

Supplement: Figure S9 — PCAF occupancy and H3K9 acetylation patterns on rRNA genes analyzed in growing HeLa cells and in cells arrested in S-phase. (A) FACS analysis on growing HeLa cells, HeLa cells arrested in G1/S with aphidicolin, HeLa cells in S-phase 4 h after release from the aphidicolin block and in S/G2 phase 6 h after release from the aphidicolin block. (B) ChIP analysis on chromatin isolated from growing HeLa cells and cells in S-phase 4 h after release from the aphidicolin block, using antibodies to PCAF and acetylated H3K9, as indicated below the x-axis. qPCR analysis was performed with primers amplifying rRNA gene promoter. The values are presented as the percentage of the input signal for each pair. Error bars represent standard deviations. p PCAF = 0.00378 (**), p RK605AA = 0.00018 (***). (TIF) [file pgen.1003397.s009.tif]

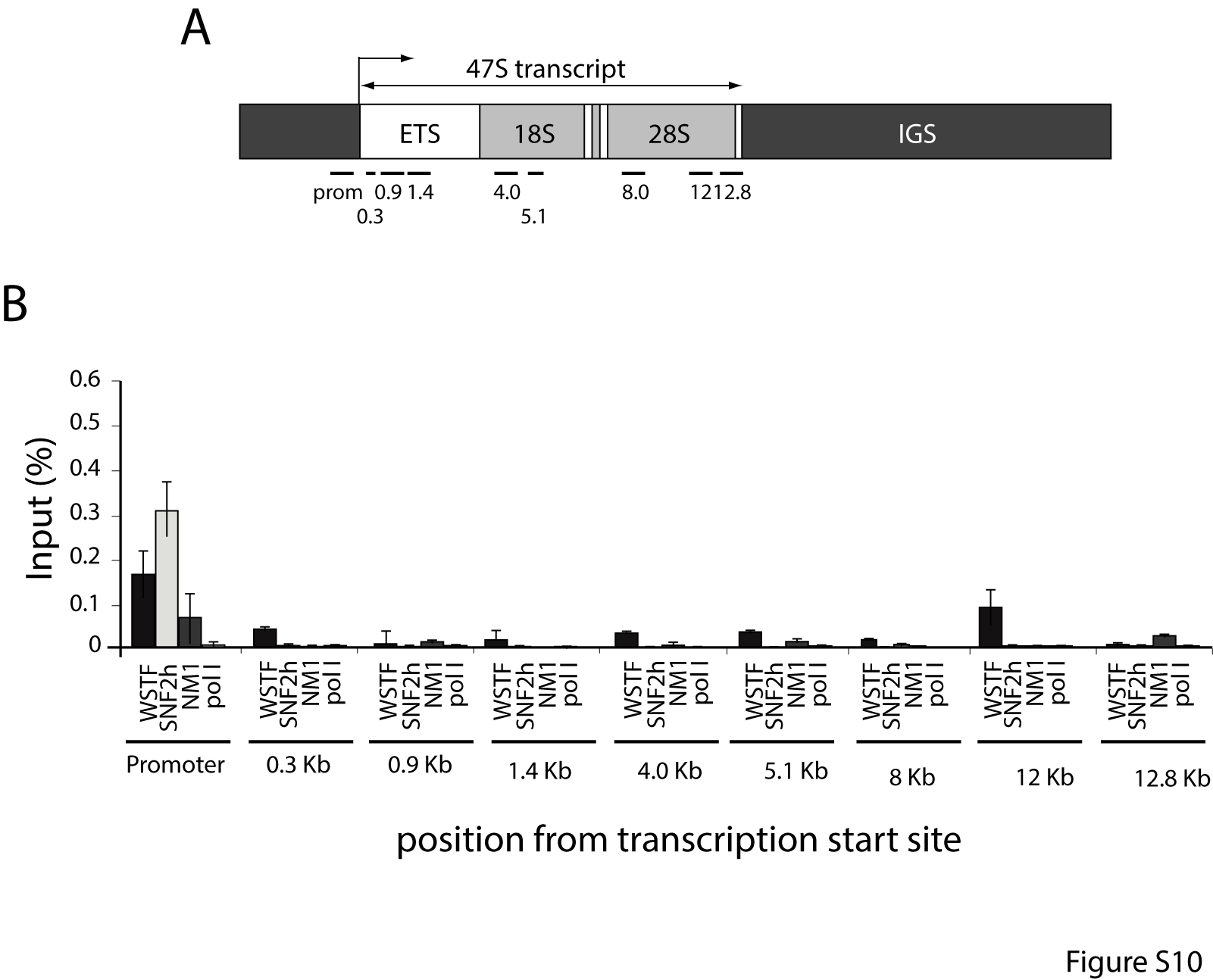

Supplement: Figure S10 — (A) Schematic representation of the human rDNA transcription unit and positions of PCR products. (B) ChIP on HeLa cells subjected to a pol I transcriptional block with actinomycin D (50 ng/ml) using the antibodies indicated below the bars. The values are presented as the percentages of the input signal for each primer pair. Error bars represent standard deviations from four separate experiments. (TIF) [file pgen.1003397.s010.tif]
