# Supplementary material for: Nuclear Myosin 1c Facilitates the Chromatin Modifications Required to Activate rRNA Gene Transcription and Cell Cycle Progression
Source: PLoS Genet. 2013 Mar 21;9(3):e1003397. doi: 10.1371/journal.pgen.1003397 (PMC3605103; doi:10.1371/journal.pgen.1003397)
Supplement: Table S1 — Sequences of primers used in the qPCR analyses of ChIP experiments. (DOC) [file pgen.1003397.s011.doc]

**Table S1**

| **rDNA region** | **Primer sequences** | **References** |
| --- | --- | --- |
| Promoter, pair 1  (prom) | Forward: 5´-GGT ATA TCT TTC GCT CCG AG  Reverse: 5´-AGC GAC AGG TCG CCA GAG GA | [1] |
| Promoter, pair 2  (prom) | Forward: 5´-AGC GAC AGG TCG CCA GAG GA  Reverse: 5´-GCG ATG GTG GCG TTT TTG G | [2] |
| 0.3 kb | Forward: 5´-TGT CAG GCG TTC TCG TCG  Reverse: 5´-GAG AGC ACG ACG TCA CCA |  |
| 45S (0.9kb) | Forward: 5´- CTC CGT TAT GGT AGC GCT GC  Reverse: 5´- GCG GAA CCC TCG CTT CTC | [1] |
| H1 (1.4kb) | Forward: 5´-GGC GGT TTG AGT GAG ACG AGA  Reverse: 5´-ACG TGC GCT CAC CGA GAG CAG | [3] |
| H4 (4kb) | Forward: 5´-CGA CGA CCC ATT CGA ACG TCT  Reverse: 5´-CTC TCC GGA ATC GAA CCC TGA | [3] |
| 5.1 kb | Forward: 5´- CAA CTT CTT AGA GGG ACA AG  Reverse: 5´- CAT GGG GAA TAA TTG CAA TC |  |
| H8 (8kb) | Forward: 5´-AGT CGG GTT GCT TGG GAA TGC  Reverse: 5´-CCC TTA CGG TAC TTG TTG ACT | [3] |
| 12kb | Forward: 5’ –CTA TCC AGC GAA ACC ACA G  Reverse: 5’ – GAT CAG AGT AGT GGT ATT TCA C |  |
| H13 (12.8kb) | Forward: 5´- ACC TGG CGC TAA ACC ATT CGT  Reverse: 5´- GGA CAA ACC CTT GTG TCG AGG | [3] |
| H27 (27kb) | Forward: 5´-CCT TCC ACG AGA GTG AGA AGC G  Reverse: 5´-CTC GAC CTC CCG AAA TCG TAC | [3] |

**References**

[1] Philimonenko VV, Zhao J, Iben S, Dingova H, Kysela K, et al (2004) Nuclear actin and myosin I are required for RNA polymerase I transcription. Nat. Cell Biol. 6: 1165–1171.

[2] Gorski SA, Snyder SK, John S, Grummt I, and Misteli T (2008) Modulation of RNA polymerase assembly dynamics in transcriptional regulation. Mol. Cell 30: 486–497.

[3] Grandori C, Gomez-Roman N, Felton-Edkins ZA, Ngouenet C, Galloway DA et al. (2005) c-Myc binds to human ribosomal DNA and stimulates transcription of rRNA genes byRNA polymerase I. Nat. Cell Biol. 7: 311–318.
