# Supplementary material for: Nuclear Myosin 1c Facilitates the Chromatin Modifications Required to Activate rRNA Gene Transcription and Cell Cycle Progression
Source: PLoS Genet. 2013 Mar 21;9(3):e1003397. doi: 10.1371/journal.pgen.1003397 (PMC3605103; doi:10.1371/journal.pgen.1003397)
Supplement: Table S2 — Sequences of primers used in the MNase experiment [4]. (DOC) [file pgen.1003397.s012.doc]

**Table S2**

| **Primer pair** | **Forward** | **Reverse** |
| --- | --- | --- |
| 42281 | 5’-CGTGCAGGTTTATGTGGG | 5’-GCATCGAGGGCTCCGGGC |
| 42370 | 5’-GTAGCTCCCGAGGCCCG | 5’-CCGGCTTAAGCAAAGGCTC |
| 42453 | 5’-CGCTCATCCTGGCCGTC | 5’-GAGACGGCGCTAGGAAAGAC |
| 42589 | 5’-GATCCTTTCTGGCGAGTCC | 5’-GGCTTTTACGAAGGCCGAG |
| 42760 | 5’-CGTGGATTCCGGAAGAGCC | 5’-GGAGGGACGAAGGCTCTC |
| 42808 | 5’-GTCCTTGGGTTGACCAGAG | 5’-GTCCACAGGCACAGGCACAG |
| 42854 | 5’-GCGATGGTGGCGTTTTTGG | 5’-CCCTCCATATAGAAAGCGAG |
| 2c | 5’-CTGACACGCTGTCCTCTGG | 5’-GGCGCGAGGACGGAACTC |
| 85c | 5’-CTAGCCGGCCGCGCTCC | 5’-CCTTCCGCCGCTCCCGG |
| 1 kb c | 5´-GGC GGT TTG AGT GAG ACG AGA | 5´-ACG TGC GCT CAC CGA GAG CAG |

**Reference**

Vintermist A, Böhm S, Sadeghifar F, Louvet E, Mansén A, et al. (2011) The Chromatin Remodelling Complex B-WICH Changes the Chromatin Structure and Recruits Histone Acetyl-Transferases to Active rRNA Genes. PLoS One 6: e19184.
